# The health impact of hazardous waste landfills and illegal dumps contaminated sites: An epidemiological study at ecological level in Italian Region

**DOI:** 10.3389/fpubh.2023.996960

**Published:** 2023-02-27

**Authors:** Lucia Fazzo, Valerio Manno, Ivano Iavarone, Giada Minelli, Marco De Santis, Eleonora Beccaloni, Federica Scaini, Edoardo Miotto, Domenico Airoma, Pietro Comba

**Affiliations:** ^1^Department of Environment and Health, Istituto Superiore di Sanità, Rome, Italy; ^2^World Health Organization Collaborating Centre for Environmental Health in Contaminated Sites, Rome, Italy; ^3^Statistical Service, Istituto Superiore di Sanità, Rome, Italy; ^4^Department of Medicine, University of Udine, Udine, Italy; ^5^Avellino Prosecution Office, Former North Naples Prosecution Office, Avellino, Italy; ^6^Fellow, Collegium Ramazzini, Bologna, Italy

**Keywords:** hazardous waste, landfills, dumps, mortality, hospitalization, cancer, low birth weight, preterm birth

## Abstract

**Background and aim:**

The implementation of idoneous management of hazardous waste, in contrast to illegal practices, is one of the environment and health priorities of the WHO. The aim of the present study, based on a collaborative agreement between the Italian National Health Institute and a Prosecution Office located in Naples North, was to evaluate the health effects of illegal landfills and burning of urban and hazardous waste in the territory of the Prosecution Office.

**Methods:**

The municipalities included in the study territory were investigated with respect to the regional population. Regression analyses were performed in the study area between four classes of an environmental municipal indicator of waste risk (MRI) previously defined, computing the relative risks (RRs) in 2–4 MRI classes, with respect to the first MRI class (the least impacted). The prevalence of reproductive outcomes and cause-specific mortality and hospitalization were analyzed in the general population and in the 0–19-year-old population using SAS software.

**Results:**

An increase of mortality and hospitalization risk in both the genders of the whole area, with respect to regional population, were found for overall all cancer cases, cancer of the stomach, the liver, the lung and the kidney, and ischemic heart diseases. An increase of mortality for leukemias in the 0-19-year-old population and in hospitalization risk for certain conditions originating in the perinatal period were observed. Correlation between MRI and the risk of mortality from breast tumors in women (MRI class 2: RR = 1.06; MRI class 3: RR = 1.15; MRI class 4: RR = 1.11) and between MRI and the risk of hospitalization from testis tumors (MRI class 2: RR = 1.25; MRI class 3: RR = 1.31; MRI class 4: RR = 1.32) were found. The hospitalization risk from breast tumors and asthma exceeded significantly in both genders of three and four MRI classes. Among the 0-19-year-old population, correlation between MRI and hospitalization from leukemias (MRI class 2: RR = 1.48; MRI class 3: RR = 1.60; MRI class 4: RR = 1.41) and between MRI and the prevalence of preterm birth (MRI class 2: RR = 1.17; MRI class 3: RR = 1.08; MRI class 4: RR = 1.25) were found.

**Conclusion:**

A correlation between health outcomes and the environmental pressure by uncontrolled waste sites was found. Notwithstanding the limitation of the study, the results promote implementing the actions of environmental remediation and the prosecution of illegal practices.

## Introduction

Mismanaged and illegal waste sites are among the principal worldwide sources of soil and groundwater pollution. In the United States, the management of waste represents the main activity causing the contamination in the areas of the National Priority List of the Environmental Protection Agency (1), including 1,334 uncontrolled hazardous waste sites in March 2022 updating (https://www.epa.gov/superfund/current-npl-updates-new-proposed-npl-sites-and-new-npl-sites, last access 15 July 2022). In Europe, 38% of the contaminated sites are characterized by municipal and industrial waste disposals (2). The World Health Organization (WHO) included hazardous waste among the main environmental risk factors for the health population in Africa (3). In three Latin American countries (Mexico, Uruguay, and Argentina), 316,703 people were estimated to be exposed to the lead released by 129 hazardous waste sites (4). The WHO estimated that only 17.4% of the e-waste produced in 2019 reached formal waste management and recycling systems (5).

Uncontrolled and poorly managed industrial and hazardous waste landfills and illegal waste dumps could release and emit a mixture of environmental contaminants, often unknown, that are potentially dangerous for the health of the population residing close to these sites (6).

The increasing body of evidence about the possible health impact of environmental contamination due to waste mismanagement prompted the WHO to recommend the implementation of sustainable waste management practices, also contrasting illegal trafficking and management, among environment and health priorities to achieve the United Nations Sustainable Development Goals (7). The evidence of the association of several health effects with exposure to hazardous waste sites has been defined as “limited”: non-Hodgkin lymphoma; cancers of the liver, the bladder, the breast, and the testis; asthma; congenital anomalies overall and of the neural tube, the urogenital, connective, and musculoskeletal systems, and low weight and preterm birth, among reproductive outcomes. This evaluation, concerning articles published through 2015, was based on more than one study reporting strong and precise results, with an overall consistent association, though the authors could not completely exclude a role of random variability, bias, and confounding factors (8).

From January 2015 to May 2022, 16 additional articles on the human health impact of hazardous waste and dumping sites, including two studies on informal workers in waste sites, the so-called “pickers,” have been published (4, 9–23) [search in PubMed and Medline: (“industrial waste” [Mesh] OR “hazardous waste” [Mesh] OR “waste disposal facilities” [Mesh] OR “electronic waste” [Mesh] OR “illegal dump^*^” [Title/Abstract]) AND (“epidemiology” [all fields] OR “mortality” [all fields])]. The articles of interest were selected by two researchers who were blinded, among the 143 articles emerged from the search, based on compliance with the inclusion criteria (epidemiological studies on humans) and the search question, in terms of population/exposure/comparators/outcomes [population: resident population; exposure: living near hazardous and electronic waste sites and illegal dumps; comparators: all comparators; outcome: all diseases/health disorders (PECO)].

The majority of the selected articles concerns reproductive and childhood health outcomes. A systematic review published in 2017 highlighted the significantly elevated risk of preterm birth (PTB) among infants born to women living near hazardous waste sites and of congenital malformations in proximity to specific waste sites (10). Increased risks of low birth weight, intrauterine growth retardation, and vector-borne diseases, such as malaria, in the population living near dumps and burning waste sites, have been reported in a more recent review (22). An increase of very preterm birth, low and very low birth weight, and stillbirth were reported among mothers exposed to contaminants released by an illegal arson of a large municipal landfill during the periconception period and the first trimester (15). A population-based case–control study (9) found an increased risk of bone tumors in children (0–14 years old) living within 2 km of hazardous waste sites, and the impact of lead released by 129 hazardous waste sites in Latin American countries was estimated to be 51,432 DALYs for mild intellectual disability in children and cardiovascular disease in adults (4). An investigation performed on the acute effects consequently to an event of illegal dumping of tons of waste into a river in Malaysia reported shortness of breath, cough, nausea, vomiting, and eye and throat irritation in school children (6–17 years old) (23). An increase in mortality for all causes, specifically for all cancers and colon–rectum, bladder, and hematological tumors, in the general population (all ages) was reported by an ecological study in residents of a municipality with landfills (13). Some studies based on self-reported symptoms in the population living close to dumpsites and mismanaged landfills in low- and middle-income countries (LMICs) reported an increase in the prevalence of diabetes (19, 20), asthma, tuberculosis and depression (20), sore throat and hypertension (19), respiratory symptoms (wheezing and frequent sneezing), and skin rashes (21). Two biomonitoring investigations performed in Italian contaminated areas by illegal waste sites were recently published. The first one concerns a subarea of the so-called “Land of Fires” in the Campania Region, which is characterized by a widespread presence of dumps and uncontrolled landfills (including waste burning sites): no correlation of persistent organic pollutants (POPs: PCBs, PCDDs, PBDEs, and PCDFs) and heavy metals blood concentration was observed with residence in the study area, but the highest values, also in comparison to the national average level, were reported in the municipality with the highest number of illegal and uncontrolled landfills (16). The importance of using private well water and consuming locally-bred eggs and beef in determining high blood levels of β-hexachlorocyclohexane (β-HCH) in the population residing within 1 km of the Sacco river, where illegal waste dumping occurred, was highlighted (18).

A special mention should be made of the articles on the health impact of electronic and electrical equipment, also known as “e-waste,” which has become an increasing problem in recent years, particularly in LMICs.

Some environmental monitoring studies observed high concentrations of heavy metals, dioxin-like compounds, and polycyclic hydrocarbons (PAHs) in e-waste sites (24–26), and some of the same compounds were also reported in blood or urine samples of the general population (27, 28), children (27, 29–34), and mothers (35, 36). Exposure to these components is reported in association with the alteration of fibrosis indicators (TGF-β and α-SMA) in the general population (27). Exposure to e-waste has been related to a high prevalence of childhood disorders: altered developmental measures (33, 35–37), neurodevelopment (30, 31, 38), behavioral disorders (38), anemia (29, 33), altered lung function (35, 37), and vascular inflammation and high blood pressure (34). In 2021, the WHO defined that prenatal and childhood e-waste exposure are significantly linked with specific birth and childhood health outcomes: impaired neurodevelopment and behavior, negative birth outcomes (including stillbirth, premature birth, shortened gestational age, low birth weight), lung functions and respiratory effects, impaired thyroid, cardiovascular and immune systems' functions, including greater vulnerability to common infections and reduced response to immunization, DNA damage, and increased risk of some chronic diseases later in life (5). The review published in the same year was consistent with the WHO report, defining “suggestive” the association between these outcomes and e-waste exposure (39).

In this context, the present article describes a study aimed at estimating the health impact of residential exposure to uncontrolled landfills and illegal dumps in Italy, based on a collaborative agreement between the Italian National Health Institute (Istituto Superiore di Sanità: ISS) and the Naples North Prosecution Office (NNPO).

**Figure 1:**
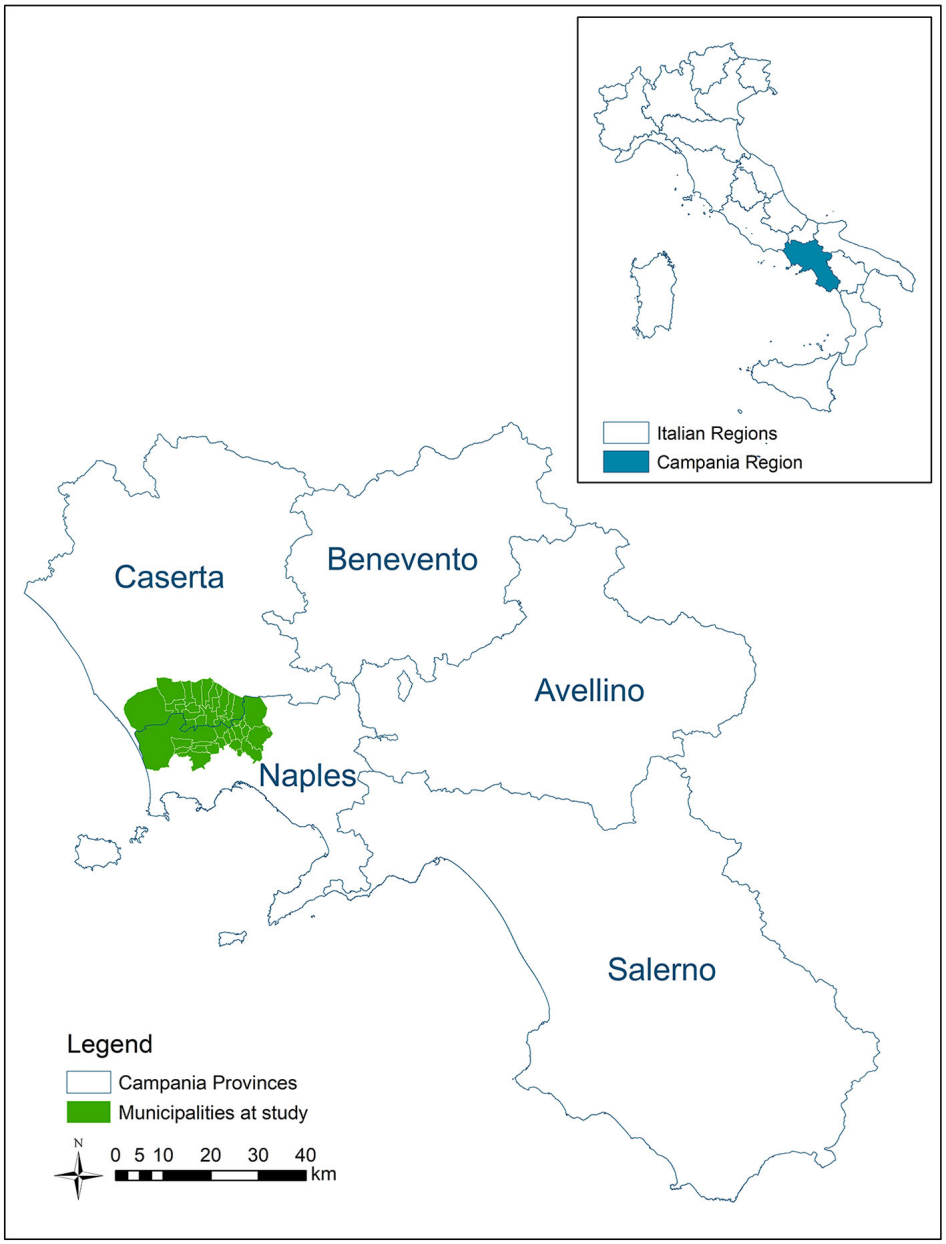
Area at study.

The study area (Figure 1) is the territory of NNPO, which includes 38 municipalities located between the Naples and Caserta provinces in the Campania Region (South Italy), and is characterized by a huge presence of waste sites (about 3,000 waste sites in 426 km^2^). Because of the environmental pressure due to the waste sites, the area is partially included among the contaminated sites of national concern for remediation. In addition, some subareas are included in the so-called “Land of Fires” national environmental emergency area, owing to illegal practices of waste open-air burning that have occurred since the 2000s. Illegal waste trafficking and mismanagement by crime organizations in the area have been documented since the end of the 1980s based on crime organization exponents' statements and judicial investigations. Industrial and urban waste, including those that are hazardous, have been illegally dumped in heaps, sunken, or buried in pits (illegal dumps), or disposed of in poorly managed landfills with no control (“uncontrolled” landfills) (40). Based on the European Legislation (Directive 91/689/EEC), transposed in Italian Legislation by means of Legislative Decree 152/06, the wastes are classified as hazardous, considering its origin, if it is known, the chemical–physical and toxicological characteristics of the substances potentially present in the waste itself. Before the cooperation agreement, both institutions had extensively investigated the area of interest. NNPO has been contrasting illegal practices of waste management since the early 1980s. ISS, in the meanwhile, had conducted a series of epidemiological studies on cancer mortality, cancer incidence, and prevalence of congenital anomalies at birth in the Provinces of Naples and Caserta in relation to waste contamination (41–44).

The first step of the collaborative study consisted in the implementation of a geo-database of the waste sites and the development of a GIS-based indicator of waste risk (40). In the study area, which is 426 km^2^ large, 2,767 waste sites, including illegal waste burning, were mapped and characterized on the basis of the environmental data and information available at the beginning of the investigation. A total of 38% of the population was estimated to be living within 100 m of one or more waste sites, areas potentially impacted by the contaminants emitted or released by the waste sites. The choice of a large buffer of 100 m around the waste site to identify the potentially impacted areas, relatively short with respect to those of 1–2 km used in other similar contexts, was due to the high density of waste sites in the study area. The data sources, including information collected by the Prosecutor through judiciary inquiries, considered the waste sites identified in the 2008–2017 period; at the beginning of the investigation, significant remediation acts have not yet been carried out. The method used to assign the index of waste risk to each municipality (municipal waste risk index: MRI) was described in the article previously published (40). A hazard risk quantification (hazard risk level: HRL) was attributed by experts' knowledge to each of the 2,767 waste sites on the basis of the information available for all sites: modality of waste disposal (i.e., illegal burning sites and dumps, controlled landfills and treatment plants, temporary storage), characteristics of the site, environmental contaminants present in the site, and type of waste. The highest level of HR was attributed to the 653 burning waste sites based on the possible contamination of all environmental media (air, soil, and water). There was no information on the duration of the fires, but the sporadic ones reported by individuals were not considered: the included sites concern arsons of waste heaps, plastic, and temporary waste storage that occurred between 2011 and 2018, as documented by law enforcement and regional institutions. To follow, no visible dumps (sunken or buried) of potentially hazardous and highly hazardous waste were considered very high-impacting waste sites. Based on the site HRL and on the estimated population residing in each impacted area (within 100 m of one or more waste sites), a municipal waste risk index (municipal risk index: MRI) was computed; the 38 municipalities were then categorized into four classes of MRI (1–low to 4–high) (details provided in the original article) (40).

The present contribution assesses the health profile of populations living in the territory of NNPO, as compared to the regional population and presents results of the regression analyses linking the risk from selected health outcomes to the municipal environmental waste risk indicator (MRI) within the study area to estimate the health impact of uncontrolled and illegal waste management in the territory of Naples North Prosecution Office jurisdiction.

In particular, cause-specific mortality and hospitalization and birth certificates in the population living in the study area were analyzed, and the possible correlation with the environmental waste risk indicator, previously elaborated, was evaluated.

## Materials and methods

**Figure 2:**
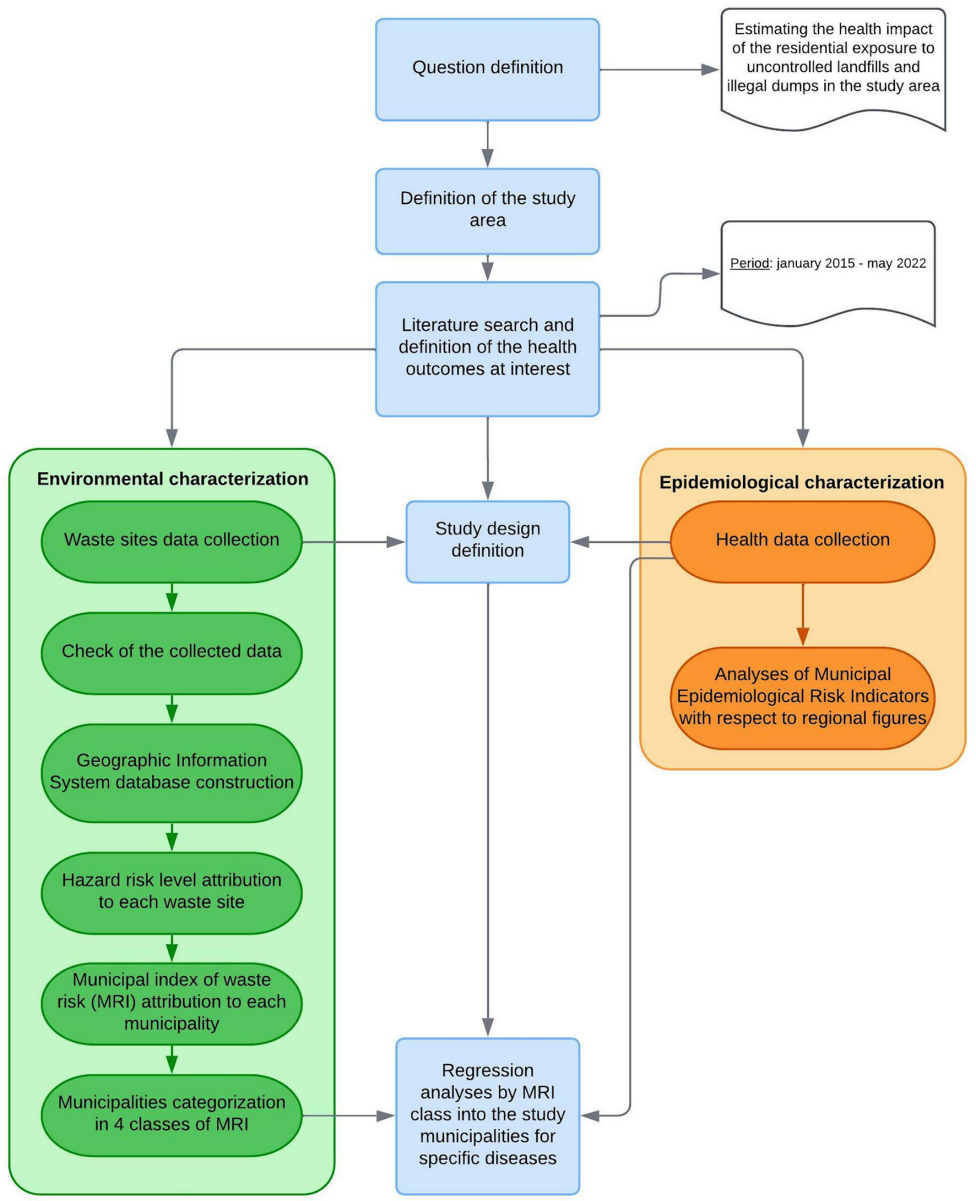
Methodology flow chart.

The sequential steps of the study are summarized in the methodology flow chart (Figure 2).

The diseases of interest for the aim of the investigation were selected *a priori*, considering the abovementioned review on the health impact of hazardous waste (8) and the toxicological literature on the contaminants reported in the waste sites of the study area.

We analyzed the municipal mortality and hospitalization database (2008–2019 period) available at the Statistics Office of the National Institute for Health, based on the Italian National Institute of Statistics (ISTAT) and Ministry of Health data, respectively. We considered the main cause reported in the death certificate and the principal diagnosis of the hospitalization discharge. For cancer diseases, a *wash-out* period up to 2001 was considered to estimate the first hospitalization, while the first hospitalization during the 2008–2019 period was considered for the other hospitalization diagnoses. For each selected disease, we analyzed the more informative outcome on the basis of the etiopathogenic characteristics.

In addition, we analyzed the birth assistance certificate (2003–2017 period) provided by the Ministry of Health to estimate the risk of low birth weight (LBW, born alive with weight <2,500 gr) and of preterm birth (PTB, born alive with gestational age <37 weeks). The analyses of the prevalence of PTB excluded twins and the analyses of LBW excluded PTBs and twins.

### Overall study area with respect to the regional population

To evaluate the health status of the population residing in the overall study area (38 municipalities combined), we computed the gender-specific standardized mortality and hospitalization ratios (SMR and SHR) for selected diseases with respect to the regional population, excluding the residents in the study area. The analyses were performed both for the general population (all ages) and for specific age classes (0–1 and 0–19 years).

For LBW and PTB, we computed the ratio of prevalence (percentage of overall born alive) in study areas *vs*. the prevalence in the referent population (Campania Region, excluding the 38 municipalities in the study).

### Regression analysis into the study area by MRI class

In the previous study, the municipalities were categorized into four MRI classes (increasing waste risk from 1 to 4 MRI classes) on the basis of environmental characterization of the waste sites and the population living within 100 m of one or more waste sites (40).

Details on the used method to compute MRI were described in the original article. The principal steps are reported further in this study. Afterward, the attribution of a hazard risk level to each waste site, following the criteria described in the Introduction section, the population living within 100 m to one or more waste sites was estimated.

To achieve this goal, the layers of the waste sites and those of the census tract sections were combined in GIS software: a new layer consisting of about 26,000 polygons was generated.

A multi-code HRL (equal to the sum of HRLs) was attributed to the areas influenced by more than one site, with an *ad hoc* procedure. The population living in the areas impacted by waste was estimated on the basis of the density of the population in the census tract where the polygon falls. For each polygon, a risk index (RI) was computed.


$$\text{RI = S}{\;}^{*}\text{ HRL}{\;}^{*}\text{ S/Sc}{\;}^{*}\;\text{P},$$

where S is the surface of the polygon, HRL is the hazard risk level index of the waste site, or the multi-code HRL of the waste sites, lying in the polygon, Sc is the surface of the census tract, P is the population residing in the census tract, S/Sc × P is the estimated population residing in the polygon, and RI is proportional to the population living in the census tract: for an inhabited census tract, the RI is equal to 0.

Subsequently, the waste risk index at the municipal level (municipal risk index: MRI) was computed, summing up the scores of all areas (polygons) comprising the municipality.


$$\begin{array}{l} MRI=\overset{n}{\underset{p=1}{\sum }}{RI}_{p}, \end{array}$$

where *p* is the number of polygons lying in the municipality and *RI*_*p*_ is the risk index of polygons lying in the municipality.

Finally, the 38 municipalities were categorized into four classes of MRI (1–low to 4–high), using Jenks' method (natural breaks) to maximize homogeneity within groups and variance between groups (40).

In the present investigation, regression analyses by MRI class into the 38 municipalities of the study area were performed for the diseases recognizing waste exposure among the risk factors with evidence defined limited (8). The relative risks (RR, 90% confidence interval) in MRI classes 2, 3, and 4 with respect to MRI class 1, composed of the municipalities less impacted by the waste sites, were computed. A generalized linear model was applied, using SAS software 9.4 version.

The analyses were performed in the general population and in the 0–19-year-old population for specific outcomes.

## Results

### Overall study area with respect to the regional population

The study area is constituted of 38 municipalities, 426 km^2^ large, with 973,509 inhabitants (2019 Census). The area is located in the Campania Region (Southern Italy), between Naples and Caserta Provinces, partially included in a contaminated site of national concern for remediation (“Domitio-flegreo e agro Aversano”) and in the so-called “Land of Fires,” because of the presence of illegal waste burning sites (Figure 1). In the area, 2,767 waste sites, including illegal waste burning (653 sites), were mapped and 38% of the population was estimated to living within 100 m of one or more waste sites (40).

Tables 1, 2 report the results of the analyses of mortality and hospitalization risks for the investigated diseases (SMRs and SHRs) in the general population living in the study area, by gender. The whole study area showed an increase in mortality and hospitalization, with respect to the regional population, in both genders, for overall malignant tumors, particularly for cancers of the stomach, the liver, the lung, and the kidney and for ischemic heart diseases. Exceeding mortality in men and women was observed also for skin melanoma and chronic liver diseases and cirrhosis. In addition, hospitalization was higher in both genders for larynx, bladder, and thyroid gland cancers, dementia, Alzheimer's disease, and acute myocardial infarction. Breast cancer was exceeding in women, both in terms of both mortality and hospitalization.

**Table 1:** Mortality in the general population in the whole area, by gender. 2008–2019 period.

**ICD-10 code**	**Mortality cause**	**Men**		**Women**	
		**Obs**	**SMR (90% CI)**	**Obs**	**SMR (90% CI)**
C00–C97	Malignant neoplasms	14,566	121 (120–123)	9,857	116 (114–118)
C16	Malignant neoplasm of stomach	918	142 (135–150)	615	138 (129–147)
C22	Malignant neoplasm of liver and intrahepatic bile ducts	1,382	142 (136–148)	766	156 (147–166)
C25	Malignant neoplasm of pancreas	613	110 (103–118)	541	104 (97–111)
C32	Malignant neoplasm of larynx	347	146 (134–159)	37	115 (88–150)
C33–C34	Malignant neoplasm of trachea, bronchus, and lung	4,706	132 (129–135)	1,274	117 (112–122)
C43	Malignant melanoma of skin	163	123 (108–140)	130	127 (110–146)
C45.0	Mesothelioma of pleura	64	99 (80–121)	25	134 (96–186)
C49	Malignant neoplasm of other connective and soft tissue	51	90 (72–113)	42	85 (66–110)
C50	Malignant neoplasm of breast	20	122 (85–176)	1,639	110 (105–114)
C61	Malignant neoplasm of prostate	846	101 (95–107)		
C62	Malignant neoplasm of testis	17	88 (59–131)		
C64, C66, C68	Malignant neoplasms of kidney, ureter, and other unspecified urinary organs	344	120 (110–131)	174	131 (116–149)
C67	Malignant neoplasm of bladder	899	130 (123–137)	167	105 (92–119)
C70–C72, D33	Malignant neoplasms of central nervous system	324	102 (93–112)	258	109 (98–121)
C73	Malignant neoplasm of thyroid gland	36	118 (90–155)	29	72 (53–97)
C81–C96	Malignant neoplasms, stated or presumed to be primary, of lymphoid, haematopoietic, and related tissue	943	101 (96–107)	763	101 (96–108)
C82–C85	Non-Hodgkin lymphomas	309	100 (91–110)	249	105 (95–117)
C91–C95	Leukaemias	418	105 (97–114)	308	95 (87–105)
C91	Lymphoid leukemia	119	95 (82–110)	87	95 (80–114)
C92	Myeloid leukemia	78	91 (75–109)	71	99 (82–121)
G12.2	Motor neuron disease	70	78 (64–95)	64	91 (74–112)
J18, J20–J22	Acute respiratory diseases	229	97 (87–109)	214	97 (86–108)
I20–I25	Ischaemic heart diseases	5,212	110 (108–113)	5,017	123 (120–126)
I21	Acute myocardial infarction	1,775	89 (86–92)	1,291	98 (94–103)
N00–N08, N17–N19	Glomerular diseases and renal failure	589	99 (93–106)	798	124 (117–131)
K71–K74	Chronic liver diseases and cirrhosis	852	117 (110–124)	825	145 (137–154)

ICD-10, International Classification of Diseases 10th revision; obs, observed cases; SMR, standardized mortality ratio; CI, confidence interval.

**Table 2:** Hospitalization in the general population in the whole area, by gender. 2008–2019 period.

**ICD-9 CM code**	**Hospitalization cause**	**Men**		**Women**	
		**Obs**	**SHR (90% CI)**	**Obs**	**SHR (90% CI)**
140–208	Malignant neoplasms	26,774	108 (107–109)	23,443	103 (102–104)
151	Malignant neoplasm of stomach	1,197	136 (130–143)	769	129 (121–137)
155	Malignant neoplasm of liver and intrahepatic bile ducts	1,634	125 (120–130)	812	134 (127–142)
157	Malignant neoplasm of pancreas	625	104 (97–111)	537	100 (93–107)
161	Malignant neoplasm of larynx	853	127 (120–135)	152	150 (131–171)
162	Malignant neoplasm of trachea, bronchus, and lung	4,535	125 (122–128)	1,391	113 (108–118)
172	Malignant melanoma of skin	631	115 (107–122)	604	106 (99–113)
163	Malignant neoplasm of pleura	147	103 (90–118)	55	99 (79–123)
171	Malignant neoplasm of connective and other soft tissue	273	95 (86–105)	218	97 (87–108)
174–175	Malignant neoplasm of female and male breast	91	139 (117–166)	6,537	99 (97–101)
185	Malignant neoplasm of prostate	2,791	86 (84–89)		
186	Malignant neoplasm of testis	496	101 (94–109)		
189	Malignant neoplasm of kidney and other and unspecified urinary organs	1,241	113 (108–119)	604	113 (105–121)
188	Malignant neoplasm of bladder	4,072	114 (111–117)	918	111 (105–117)
191–192	Malignant neoplasm of central nervous system	623	94 (88–100)	508	92 (86–99)
193	Malignant neoplasm of thyroid gland	488	107 (100–116)	1,469	104 (100–109)
200–208	Malignant neoplasm of lymphatic and hematopoietic tissue	2,393	101 (98–104)	2,006	102 (98–106)
200, 202	Non-Hodgkin lymphomas	1,190	102 (97–106)	995	103 (98–109)
204–208	Leukemias	835	103 (97–109)	638	104 (98–111)
204	Lymphoid leukemia	380	99 (91–107)	282	102 (93–113)
205	Myeloid leukemia	447	105 (97–113)	333	97 (89–106)
250	Diabetes mellitus	3,499	86 (83–88)	3,025	91 (88–94)
290.0, 290.4, 331.1–331.2	Dementias	449	133 (123–143)	545	123 (115–132)
331.0	Alzheimer's disease	156	90 (79–102)	264	87 (78–96)
332	Parkinson's disease	455	85 (79–92)	324	82 (75–90)
335.2	Motor neuron disease	142	92 (80–105)	112	103 (88–121)
460–466, 480–487	Acute respiratory diseases	12,113	82 (81–83)	9,319	81 (79–82)
493	Asthma	2,875	88 (86–91)	2,432	85 (82–88)
410–414	Ischemic heart disease	23,902	102 (101–103)	11,079	109 (107–111)
410	Acute myocardial infarction	11,749	115 (113–117)	5,204	124 (121–127)
580–586	Nephritis, nephrotic syndrome, nephrosis, renal failure included	5,115	95 (93–97)	4,354	105 (102–107)
571	Chronic liver disease and cirrhosis	5,275	92 (90–94)	3,663	101 (98–103)

ICD-9 CM, International Classification of Diseases 9th revision Clinical Modification; obs, observed cases; SHR, standardized hospitalization ratio; CI, confidence interval.

The analyses focusing on pediatric-adolescent subpopulations showed an increase in mortality for leukemias in the 0–19-year-old population and in hospitalization for certain conditions originating in the perinatal period (Tables 3, 4).

**Table 3:** Mortality in the whole area, in 0–19 age class, males and females combined. 2008–2019 period.

**Age class**	**ICD-10 code**	**Mortality cause**	**Obs**	**SMR (90% CI)**
**0–19 years**				
	A00–T98	All causes	777	94 (89–100)
	C00–D48	All neoplasms	96	99 (84–117)
	C70–C72, D33	Malignant neoplasms central nervous system	17	84 (56–125)
	C81–C96	Malignant neoplasms of lymphoaematopoietic system	29	114 (84–154)
	C91–C95	All leukaemias	26	141 (102–195)
	C49	Malignant neoplasm of other connective and soft tissue	2	49 (16–148)
**0–1 year**				
	A00–T98	All causes	423	97 (89–105)
	C00–D48	Neoplasms	7	135 (73–248)
	C70–C72, D33	Malignant neoplasms of central nervous system	0	
	C81–C96	Malignant neoplasms of lymphoaematopoietic system	1	135 (30–603)
	P00–P96	Certain conditions originating in the perinatal period	241	95 (86–106)

ICD-10, International Classification of Diseases 10th revision; obs, observed cases; SMR, standardized mortality ratio; CI, confidence interval.

**Table 4:** Hospitalization in the whole area, in 0–19 age class, males and females combined. 2008–2019 period.

**Age class**	**ICD-9CM code**	**Hospitalization cause**	**Obs**	**SHR (90% CI)**
**0–19 years**				
	460–466; 480–487	Acute respiratory diseases	11,206	77 (76–78)
	493	Asthma	3,819	94 (92–97)
	580–586	Nephritis, nephrotic syndrome, and nephrosis	711	104 (98–111)
**0–1 year**				
	760–779	Certain conditions originating in the perinatal period	10,189	101 (100–103)

ICD-9 CM, International Classification of Diseases 9th revision Clinical Modification; obs, observed cases; SHR, standardized hospitalization ratio; CI, confidence interval.

The prevalence of PTB and LBW was significant higher in the whole area with respect to the regional population (Table 5).

**Table 5:** Prevalence of preterm and low birth weight, in the whole area. Males and females combined. 2013–2017 period.

	**Obs**	**% obs/born alive**	**RP (90% CI)**
Preterm birth^*^	2,870	3.71	106 (102–110)
Low birth weight^**^	1,551	6.42	108 (103–113)

Obs, observed cases; RP, ratio of prevalence; CI, confidence interval; ^*^excluding twins; ^**^excluding preterm birth and twins.

### Regression analysis into the study area by MRI class

The distribution of municipalities and population by MRI class is reported in Supplementary Table 1.

Tables 6, 7 show the RR of the mortality and hospitalization, respectively, by MRI class, using class 1 (the municipalities lowest impacted by waste) as a reference, and gender.

**Table 6:** Mortality, 2008–2019. Relative risk (RR), by gender and class of municipal environmental indicator of waste risk (MRI).

**Diseases**	**MRI class 1**		**MRI class 2**		**MRI class 3**		**MRI class 4**	
	**Men**	**Women**	**Men**	**Women**	**Men**	**Women**	**Men**	**Women**
	**RR**	**RR**	**RR (90% CI)**	**RR (90% CI)**	**RR (90% CI)**	**RR (90% CI)**	**RR (90% CI)**	**RR (90% CI)**
Malignant tumor (MT) of liver	1	1	1.17 (1.05–1.30)	1.16 (1.00–1.35)	0.99 (0.88–1.12)	1.17 (1.00–1.38)	0.91 (0.79–1.06)	1.03 (0.85–1.25)
MT of breast	1	1	1.19 (0.48–2.99)	1.06 (0.95–1.17)	1.05 (0.37–2.95)	1.15 (1.03–1.28)	1.08 (0.35–3.35)	1.11 (0.98–1.25)
MT of testis	1		1.32 (0.45–3.73)		1.76 (0.62–5.00)		0.91 (0.23–3.61)	
MT of bladder	1	1	0.87 (0.75–1.00)	0.73 (0.52–1.03)	1.00 (0.86–1.16)	1.26 (0.92–1.73)	1.18 (1.00–1.39)	0.81 (0.53–1.24)
Non-Hodgkin lymphoma	1	1	1.03 (0.81–1.32)	1.12 (0.87–1.44)	1.49 (1.17–1.89)	0.72 (0.52–0.98)	1.06 (0.78–1.42)	0.95 (0.68–1.31)

CI, confidence interval.

**Table 7:** Hospitalization, 2008–2019. Relative risk (RR), by gender and class of municipal environmental indicator of waste risk (MRI).

**Diseases**	**MRI class 1**		**MRI class 2**		**MRI class 3**		**MRI class 4**	
	**Men**	**Women**	**Men**	**Women**	**Men**	**Women**	**Men**	**Women**
	**RR**	**RR**	**RR (90% CI)**	**RR (90% CI)**	**RR (90% CI)**	**RR (90% CI)**	**RR (90% CI)**	**RR (90% CI)**
Malignant tumor (MT) of liver	1	1	1.07 (0.97–1.18)	1.14 (0.99–1.31)	0.95 (0.85–1.06)	1.12 (0.96–1.31)	0.82 (0.72–0.94)	0.74 (0.61–0.91)
MT of breast	1	1	1.48 (0.88–2.48)	1.02 (0.97–1.07)	2.61 (1.62–4.21)	1.07 (1.01–1.13)	2.62 (1.56–4.37)	1.05 (0.99–1.12)
MT of testis	1		1.25 (1.03–1.51)		1.31 (1.07–1.61)		1.32 (1.06–1.65)	
MT of bladder	1	1	0.94 (0.88–1.01)	0.92 (0.80–1.06)	0.99 (0.92–1.06)	1.20 (1.04–1.38)	0.93 (0.86–1.01)	1.03 (0.87–1.22)
Non-Hodgkin Lymphoma	1	1	0.95 (0.83–1.07)	1.02 (0.89–1.16)	1.10 (0.97–1.25)	0.94 (0.82–1.09)	1.07 (0.92–1.23)	1.02 (0.87–1.19)
Asthma	1	1	0.96 (0.90–1.05)	1.00 (0.91–1.09)	1.15 (1.06–1.25)	1.28 (1.17–1.40)	1.28 (1.17–1.40)	1.23 (1.11–1.35)

CI, confidence interval.

The mortality for breast and liver tumor was higher in female subjects of MRI classes 2, 3, and 4, with lower confidence interval values between 0.85 and 1.03; the mortality rate for bladder cancer was higher in men living in MRI class 4 (Table 6).

The hospitalization rate for breast cancer was higher in men and women living in MRI classes 2 (with lower CI limits <1), 3, and 4; in MRI classes 3 and 4, the hospitalization rate for asthma also increases. Exceeding hospitalization for testis cancer was observed in all MRI classes 2–4 with respect to class 1 (Table 7).

Table 8 shows the results of the hospitalization regression analyses in the 0–19-year-old population, and Table 9 reports the RR of PTB and LBW. Among the 0–19-year-old population, the hospitalization rate for all leukemias was higher in MRI classes 2–4, for asthma in the last two classes (MRI classes 3 and 4), and for acute respiratory diseases in the class most impacted by waste (MRI class 4) (Table 8). No increase of LBW risk was detected by MRI class, meanwhile the risk of PTB exceeds in MRI classes 2–4 (Table 9).

**Table 8:** Hospitalization, 2008–2019. Zero to nineteen years old. Males and females combined. Relative risk (RR), by class of municipal environmental indicator of waste risk (MRI).

**Causes**	**MRI class 1**	**MRI class 2**	**MRI class 3**	**MRI class 4**
	**RR**	**RR (90% CI)**	**RR (90% CI)**	**RR (90% CI)**
All malignant tumors	1	1.16 (1.00–1.34)	1.14 (0.97–1.33)	0.90 (0.75–1.08)
Leukemias overall	1	1.48 (1.08–2.03)	1.60 (1.15–2.23)	1.41 (0.98–2.02)
Acute respiratory diseases	1	1.02 (0.98–1.07)	0.94 (0.90–0.99)	1.19 (1.14–1.25)
Asthma	1	0.98 (0.92–1.05)	1.18 (1.10–1.27)	1.31 (1.21–1.41)

CI, confidence interval.

**Table 9:** Prevalence at birth, 2013–2017. Males and females combined. Relative risk (RR), by Class of municipal environmental indicator of waste risk (MRI).

	**MRI class 1**	**MRI class 2**	**MRI class 3**	**MRI class 4**
	**RR**	**RR (90% CI)**	**RR (90% CI)**	**RR (90% CI)**
Low birth weight^*^	1	0.94 (0.84–1.05)	1.00 (0.89–1.13)	1.01 (0.89–1.14)
Preterm birth^**^	1	1.17 (1.08–1.27)	1.08 (0.99–1.18)	1.25 (1.14–1.37)

CI, confidence interval; ^*^excluding twins; ^**^excluding preterm birth and twins.

Figures 3–5 show the forest plots of the main results; all forest plots of the regression analyses are reported in Supplementary Figures 1–10.

**Figure 3:**
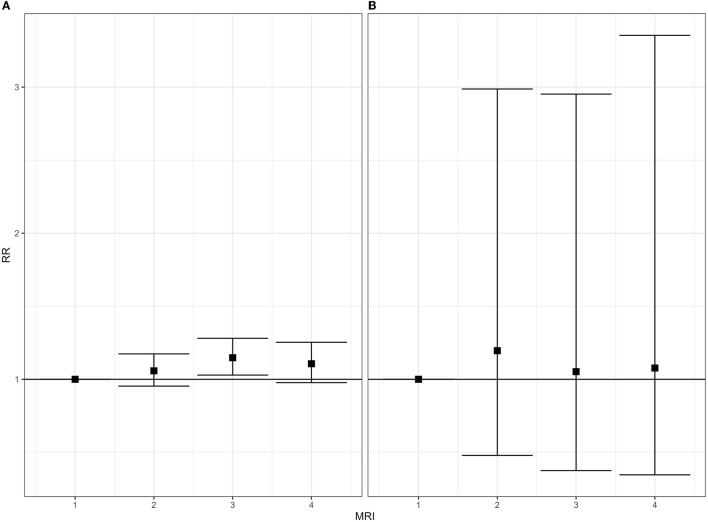
Mortality for malignant tumor of breast, 2008–2019. Relative risk (RR), by gender and class of municipal environmental indicator of waste risk (MRI). **(A)** Women; **(B)** men.

**Figure 4:**
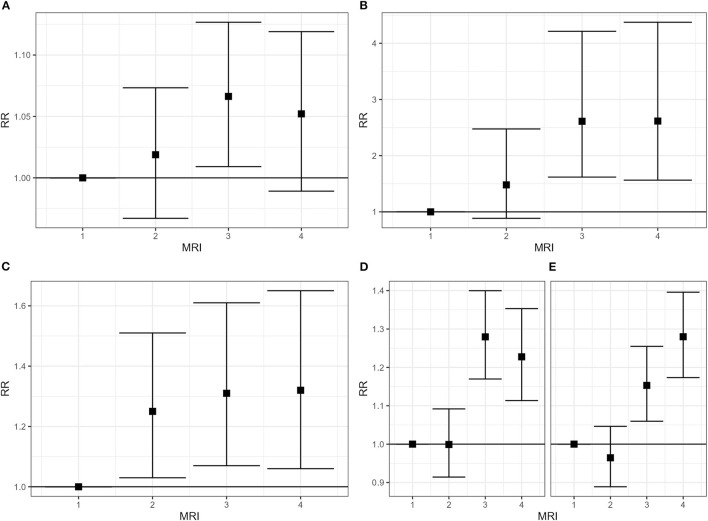
Hospitalization for malignant tumor of breast [**(A)** women, **(B)** men] and testis **(C)** and for asthma [**(D)** women, **(E)** men], 2008–2019. Relative risk (RR), by gender and class of municipal environmental indicator of waste risk (MRI).

**Figure 5:**
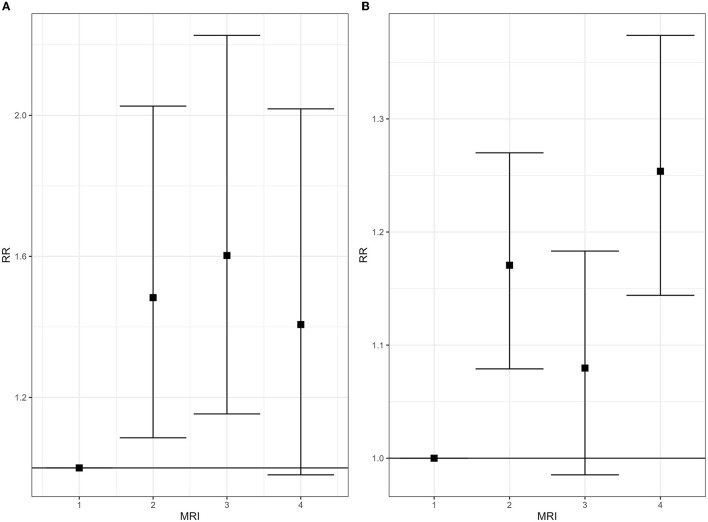
Zero to 19-year-old people. Hospitalization for leukemias, 2008–2019 **(A)** and prevalence of preterm birth, 2013–2017 **(B)**. Males and females combined. Relative risk (RR), by class of municipal environmental indicator of waste risk (MRI).

## Discussion

**Figure 6:**
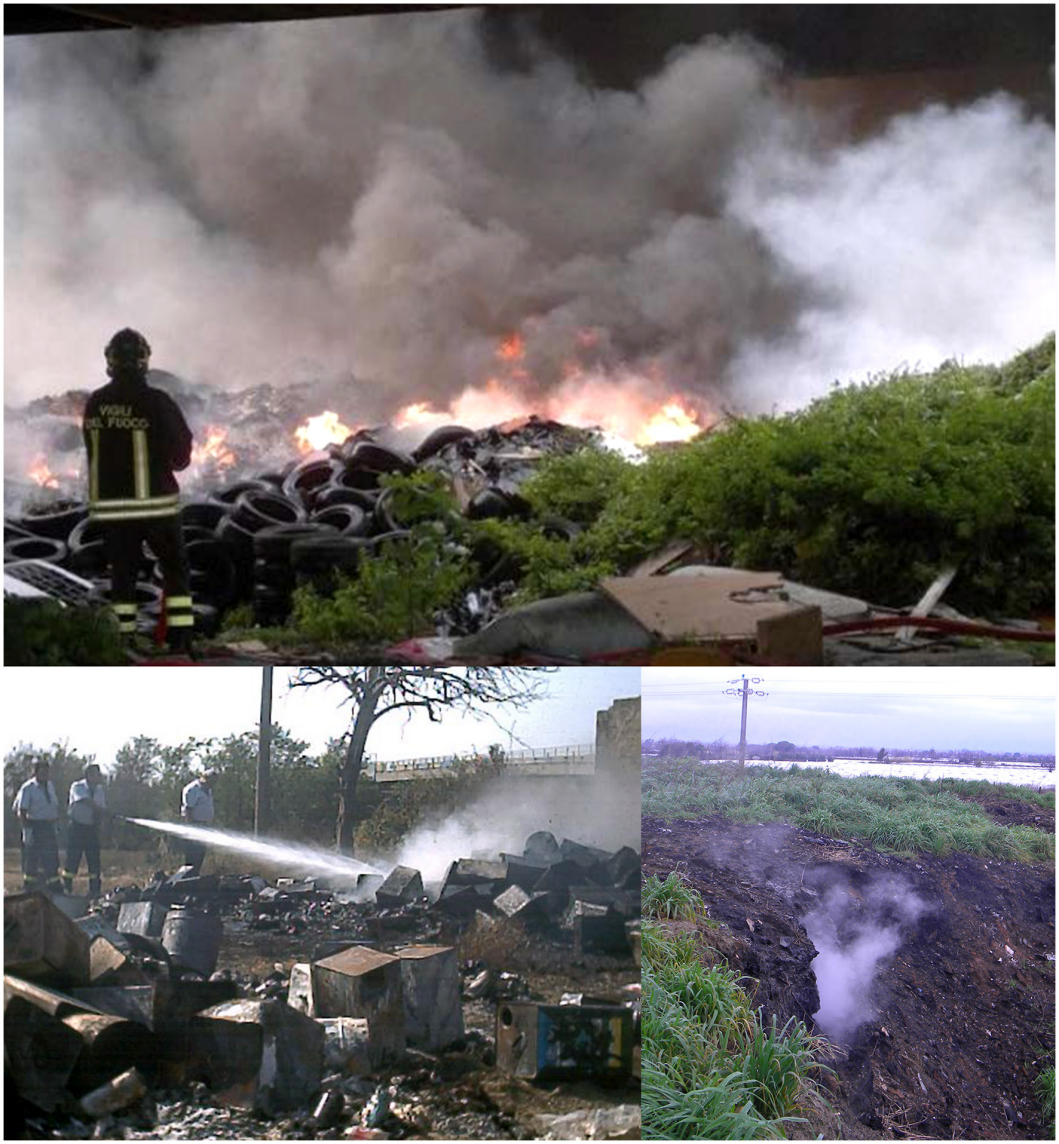
Some illegal waste dumping and burning sites in the study area.

The study area was characterized by a huge presence of waste sites (2,767 waste sites in 426 km^2^) and illegal practices of waste management (characterizing ~90% of the waste sites) that occurred in the area since the early 1980s and was documented to be present in the 2008–2017 period (Figure 6). At the beginning of the present investigation, no significant environmental remediation actions have been performed. The analyses of the health profile of the population residing in the study area show some relevant criticalities as compared to the general population of the Campania Region. Most of the excesses are, moreover, detected in both genders, supporting the role of environmental exposures.

The present investigation shows a correlation, at the municipal level, between the indicator of the environmental risk impact of the waste site (MRI) and specific health outcomes: breast and testis cancers and asthma in the general population, leukemias in the 0–19-year-old subpopulation, and the prevalence of preterm birth. The municipalities belonging to the highest MRI classes (classes 3 and 4) are characterized by illegal and uncontrolled dumps of hazardous waste, including sites where illegal waste burning occurred. Moreover, as above mentioned, in the study area significant environmental cleanup acts have not been carried out, at the beginning of the present investigation.

Some further considerations are needed in order to interpret the results.

The ecological study design at the municipal level does not allow inferring risks at the individual level but could represent a useful indicator of risks playing at the population level to identify appropriate interventions for public health (45). The assessment of exposure based on residence at the municipal level may cause a bias in the estimates, which, causing non-differential exposure misclassification, results in an underestimation of the risks (46); this issue has been addressed by several authors (47, 48), and Jurek et al. advised the use of sensitivity analysis to evaluate the measures of underestimation if local data are available (49). However, it should also be considered that the municipal environmental waste risk indicator (MRI) was built considering the populations living in the census tracts near the waste sites (40).

The regression analysis was performed among municipalities included in an area extensively impacted by waste sites, where increases in mortality rate and hospitalization for some outcomes, with respect to the regional reference, were detected.

Spatial autocorrelation between the analyzed municipalities was not taken into account, considering that the whole area is highly impacted by waste sites. This assumption could entail bias in the estimations (50); nevertheless, the present investigation aimed to analyze the risk of health outcomes as a function of the environmental indicator, highlighting the individual municipalities with higher levels of criticality.

Some biomonitoring investigations have been performed in the so-called “Land of Fires” (51–54), which includes our study area. The medium concentrations of PCB and dioxin-like agents in cow's and mother's milk were consistent with the national values, detecting individual high values in specific subareas, in some cases characterized by uncontrolled and illegal dumps and burning waste sites (50–53). A more recent study mentioned in the Introduction paragraph did not observe an association between POPs (PCBs, PCDDs, PBDEs, and PCDFs) and heavy metals blood concentrations with residence in the “Land of Fires,” but the highest values were observed in the municipality with the highest presence of waste sites (16), which coincided with one of the municipalities included in the highest MRI class in the present investigation.

Class 1 of MRI, used as a reference in the regression analyses, includes municipalities with an ascertained impact of waste sites, even if lower than the other ones. The analyses of this class, when compared to the regional population, showed an increase in both genders of mortality and hospitalization for liver and bladder cancer as well as of mortality from breast tumor; in addition, the prevalence of LBW was higher than expected (Supplementary Tables 2–4). The choice of this reference class, not to be considered as unexposed, was due to data availability and could be a limitation of the study design; however, this is expected to increase the likelihood of the exceeding risks observed in municipalities with higher MRI values.

Because of the unavailability of cancer incidence data, we analyzed the occurrence of oncological diseases through the hospital discharge records. The limitation of the use of these data to estimate the incidence of cancers is largely documented, and the results represent the risk of hospitalization for the considered tumors, even if the *wash out* period used in the selection of the hospital discharge records for these diseases could reduce the bias of the estimates. In addition to cancer registries data, which are the gold standard for assessing cancer incidence in a population, hospital discharge records could be useful in the active search for cancer cases (55). An integration of mortality and hospital discharge data with those of the cancer and congenital anomalies registries is, therefore, advisable, and an evaluation of the feasibility of further study developments is ongoing.

In addition, we did not have information on any waste site located outside the study area, and an underestimation of the waste sites' impact could affect the neighboring municipalities in particular.

The present investigation aimed to highlight the waste sites with a possible health impact on the population. In the analyses, we did not consider other risk factors because of the study design and the availability of data. The investigated diseases, even if selected on the basis of the evidence of association with exposure to substances released by the waste sites, have a multifactorial etiology, and the exposure to waste sites could concur with their occurrence. However, the regression analysis was performed among populations living in the restricted study area, likely similar in terms of socio-economic status, access to health services, environmental exposures, and lifestyles. Nevertheless, residual effects of these risk factors and of other covariates cannot be ruled out.

In particular, we found a correlation between the environmental waste risk indicator (MRI) and breast cancer mortality in women and hospitalization in both genders. The occurrence of male breast cancer is a very rare event. Breast cancer is associated with sufficient evidence with exposure to alcoholic beverages, estrogen–progesterone therapies and diethylstilbestrol, x-rays, and gamma radiation; limited evidence has been found for the association with dioxins, tobacco smoking, estrogen menopausal therapy, shift work, and exposure to PCBs (56). In addition, the excess of testicular cancer in hospitalization analysis recognizes some of the same risk factors as breast cancer, such as exposure to endocrine disruptor chemicals (EDCs: heavy metals, POPs) (57, 58). Previous biomonitoring studies performed in the same territory reported high levels of POPs and heavy metals in subareas with hazardous waste sites (51–54). The evidence of the association of breast and testis cancers with exposure to hazardous waste sites was defined as limited by the systematic review published in 2017 (8).

The hospitalization risk from asthma was significantly higher in the highest MRI classes (classes three and four). An increase in asthma was reported in the population living in the atmospheric pollutant areas. The emission of airborne pollutants by waste sites was documented (59, 60), and an increase in asthma and respiratory diseases were related to the residence near hazardous waste sites (60); in addition, in the study area, waste burning acts were largely documented.

Particular attention has to be paid to the increased risks in pediatric–adolescent subpopulations. As compared to adults, in fact, children, in general, experience higher exposure to environmental agents due to their activity patterns, behavior and physiological characteristics, and immaturity of organs and systems (https://www.epa.gov/children). Moreover, children spend more time outdoors and have higher respiratory rates. They also play close to the ground, potentially increasing their contact with polluted soils (61, 62). At the same time, children neither are usually exposed to many lifestyle factors like adults nor do they experience occupational exposures, at least in most high-income countries, such as Italy. Therefore, a stronger effect and fewer confounders are expected in children living in our study area compared to the adult population, making the detected exceeding risk as “sentinel events” to be futher attentioned. This is the case of the observed increase of hospitalization for leukemia, asthma, and acute respiratory diseases in the MRI classes most impacted by waste, which supports the hypothesis of possible environmental exposure to air pollutants among children. In particular, hospitalization from leukemias is in excess in all MRI classes most impacted by the waste sites. An increase in hematological diseases were related to the residence of hazardous waste sites containing benzene (59), and childhood leukemia has been found to be associated with residential proximity to industrial plants involved in the hazardous waste sector (63).

The high risk of prevalence of preterm birth (PTB), observed in all MRI classes, with respect to MRI class 1, was related to the mother's environmental exposure to waste sites in the gestational period (10), and the evidence of the association was limited (8). Socio-demographic factors, such as ethnicity, older age, low education levels, and smoking of the mothers, were also reported as risk factors for PTB (64). The excess of PTB is of particular interest, considering that it could represent a risk for disorders and health outcomes in adult life. PTB is a major cause of death since complications arising from these adverse reproductive outcomes are the single largest direct cause of neonatal deaths, and after pneumonia, it is the second most common cause of children under-5 years deaths (65). Effects of preterm birth on a long-term scale are documented in some reviews showing a significantly increased risk for altered cardiovascular and renal functions in young adulthood (66), higher blood pressure (67, 68), and several components of the metabolic syndrome and cardiovascular disease in adult life (69).

To correctly understand the meaning of the present study, it can be helpful to examine a few points, also bearing in mind the abovementioned limitations.

The last two decades have witnessed a growing interest in the international scientific community and of the WHO (specifically of the WHO Regional Office for Europe) in the health impact of inappropriate, if not openly illegal, methods of waste management. The most important event in this frame has been the inclusion of the topic “Waste disposal, management and trafficking and contaminated sites” among the priorities of the Declaration of the Sixth Ministerial Conference on Environment and Health of the European Region of the WHO held in Ostrava (Czech Republic) on 13–15 June 2017 (7). The inclusion of the notion of waste trafficking clearly shows the underlying relevance of the criminal world in this domain. In contrast, this phenomenon implies a strong synergy between health and judicial authorities.

In this context, one pivotal issue is to estimate the health impact of illegal waste disposal procedures. This is a most critical question because it is well-known that epidemiological studies of environmental factors produce valuable findings in terms of public health because they encompass valid procedures for exposure assessment. In this domain, though, exposure assessment is difficult because, by definition, the criminal organizations work in secret and hide as much as possible the location of the dumping sites (in addition, obviously, their specific chemical contamination). Epidemiology, being an observational, non-experimental discipline, requires the adoption of highly validated protocols to concur to the detection of causal webs between environmental exposures and health impacts [for an overview of these items, among others (47), refer to (70–73)].

When epidemiological issues are brought in the Courts, the complexity of causal evaluations increases, especially because the object of epidemiology is population health, while the issues of both toxic tort litigations and criminal prosecution concern the health of specific individuals, plaintiffs, or ascertained victims [see, among else, (74–78) references]. With respect to causal links that are well-assessed in scientific terms, such as the inhalation of asbestos fibers and the occurrence of pleural mesothelioma, doubts about biological mechanisms of action can lead to unexpected absolutions, as discussed by the Italian Association of Epidemiology in a recent position article (79).

In light of the abovementioned evidence, the purpose of the present study consists in to confirm or refute the hypothesis of a correlation between the GIS-based indicator of waste risk and the occurrence of excess cases of different diseases aggregated at the municipality level. This observation may be helpful for setting priorities for environmental cleanup with particular care for areas where indicators of children and adolescents' health are more critical.

The current limitations in our knowledge may impair the search for sufficient evidence of an association between exposure to complex chemical cocktails of pollutant agents and a wide range of adverse health outcomes. The same limitations, however, do not impede us from using the findings of the present study to guide appropriate policies on the study territory and, given the consistency of the results reported in the literature, in similar contexts. Special attention should be given to the most vulnerable population subgroups in the frame of a precautionary approach.

To reduce environmental exposure, through the contrast of illegal waste mismanagement and trafficking, the implementation of environmental remedial actions and of safe waste management is among the priority prevention acts recommended by the WHO (7). The implementation of a circular economy, with the reduction of waste production and the increase of waste reuse and recycling, seems particularly urgent at both the local and global levels.

Based on recent estimates (2020 https://www.isprambiente.gov.it/it/pubblicazioni/rapporti/rapporto-rifiuti-urbani-edizione-2021), in the Campania Region, the separate collection of waste concerns 54% of the urban waste (~2.5 million tons); in Naples and Caserta provinces (that include the study area), the percentage is similar: 48 and 54%, respectively. Moreover, about 50,000 tons of urban waste are managed in landfills outside the region, and 1% in regional landfills. In terms of hazardous waste, ~8 million tons are produced at the regional level, with 75% being recovered and the remaining 25% being heat treated. Nevertheless, uncontrolled and illegal waste dumping and burning of both urban and hazardous waste continue to occur.

These actions require measures by judiciary authorities, in terms of repression, and by administrative authorities, in terms of prevention (80). The international trade of waste, in particular of hazardous waste from industrialized to low-middle income countries (LMCIs) requires global efforts to contrast illegal acts and to control the respect of International Agreements, such as the “Basel Convention on the Control of Transboundary movements of hazardous waste and their management” and the related regulations. These efforts, at the global level, are particularly compelling, also in light of the more recent articles on the population living near waste sites and the informal workers in waste management, often children and women, in LMCIs.

In addition, healthcare and assistance plans should be implemented in these areas, with special attention paid to maternal and pediatric health and oncological diseases. The achievement of health assistance and prevention acts is strongly related to the participation of the local communities and communication plans involving public institutions and stakeholders (81).

The complexity of these contexts requires collaboration, at the global and local levels, between all institutions and organizations, including non-governmental organizations (NGOs) and citizen committees (80).

Notwithstanding the need to implement the abovementioned acts, further additional research on this issue could increase our knowledge to better point out the more appropriate actions. The majority of the published articles concern ecological studies, such as the present, and often this study design is the only possible choice, considering the huge impacted areas (80). The limitations of these studies, in order to test hypotheses of the association disease/risk factor, have been mentioned earlier. Epidemiological investigations at the individual level and human biomonitoring studies could provide useful information on the exposure and the possible biological mechanisms. The mixture of chemicals present in these sites, often unknown, make critical the development and the informativeness of these studies (80). In this regard, some articles have addressed the complexity in assessing exposure and impact of waste of industrial origin, with particular attention to the innovative exposome approach in relation to multi-route and multi-pathway exposure (82–84). In addition, some health outcomes, recently highlighted in people exposed to hazardous waste, such as diabetes, neurological and cognitive development, and physical growth, deserve further particular attention and specific focus.

Finally, the present study represents a particular example of a collaborative approach between institutions with different, though complementary, mandates: a national public health institute, in charge of identifying the health effects of exposure to environmental risk factors to identify idoneous primary preventive actions, including environmental remediation; a Prosecution Office with a specific mission to contrast and prosecute illegal waste trafficking and mismanagement in areas with documented hazardous waste contamination. The combination of the two approaches appears to be of particular interest, considering the large worldwide diffusion of illegal waste practices and transboundary trade, concerning, in particular, LMICs. The adopted investigation procedure and epidemiological methods, notwithstanding the abovementioned limitations, could represent a useful approach to deal with areas highly contaminated by an unknown mixture of toxic contaminants from several point sources.

## Conclusion

A correlation between illegal waste sites and specific diseases was observed in an area highly affected by waste sites. In particular, mortality from breast cancer in women and hospitalization from testis cancer were found to be correlated with the environmental municipal waste risk index. The hospitalization from breast cancer and asthma exceeded in both genders in the municipalities most impacted by waste sites. Among 0–19-year-old people, a positive correlation with the risk index was found for hospitalization from leukemias and for the prevalence of preterm birth.

The present results confirm that waste mismanagement, in particular of hazardous waste, could represent a health risk for the population. The implementation of policies for environmental remediation of the sites, the contrast of illegal and unsafe waste management and trafficking, and the implementation of a virtuous waste circular economy are warranted at the local and global levels.

## Data availability statement

The data analyzed in this study is subject to the following licenses/restrictions: the analysis of the data used in this study complies with the European General Data Protection Regulation (EU GDPR 2016/679) which authorized the processing of personal data relating to hospital discharge forms and causes of death by ISS and other public institutions for reasons of public interest in public health. Written consent for participation was not required for this study, in accordance with national legislation and institutional requirements. Requests to access these datasets should be directed to GM, giada.minelli@iss.it.

## Ethics statement

Ethical review and approval was not required for the study of human participants in accordance with the local legislation and institutional requirements. Written informed consent from the patients/participants was not required to participate in this study in accordance with the national legislation and the institutional requirements.

## Author contributions

LF, PC, and II were involved in the conception and design of the study and in data interpretation. LF was involved in writing the original draft preparation, review, and revision of the manuscript. PC and II were involved in reviewing the manuscript. VM and GM were involved in data collection, data analysis, and manuscript review. EB, FS, and MD were involved in data collection and manuscript review. EM was involved in writing the original draft preparation and reviewed the manuscript. DA was involved in the conception of the study and reviewed the manuscript. All authors have read and agreed to the final manuscript.

## References

[1] Pohl HR Tarkowski S Buczynska A Fay M De Rosa CT. Chemical exposures at hazardous waste sites: experiences from the United States and Poland. Environ Toxicol Pharmacol. (2008) 25:283–91. 10.1016/j.etap.2007.12.005 21783864

[2] European, Environment Agency. Progress in Management of Contaminated Sites in Europe. Available online at: https://www.eea.europa.eu/data-and-maps/indicators/progress-in-management-of-contaminated-sites-3/assessment (accessed July 15, 2022).

[3] McCormack VA Schüz J. Africa's growing cancer burden: environmental and occupational contributions. Cancer Epidemiol. (2012) 36:1–7. 10.1016/j.canep.2011.09.005 21996568

[4] Caravanos J Carrelli J Dowling R Pavilonis B Ericson B Fuller R. Burden of disease resulting from lead exposure at toxic waste sites in Argentina, Mexico and Uruguay. Environ Health. (2016) 15:72. 10.1186/s12940-016-0151-y 27339191 PMC4918194

[5] World Health Organization. Children and Digital Dumpsites: E-waste Exposure and Child Health. Geneva: World Health Organization (2021). Available online at: https://apps.who.int/iris/rest/bitstreams/1350891/retrieve (accessed July 15, 2022).

[6] World Health Organization. Waste and Human Health: Evidence and Needs. WHO Meeting Report 5-6 November, Germany. WHO Regional Office for Europe (2016). Available online at: https://www.euro.who.int/__data/assets/pdf_file/0003/317226/Waste-human-health-Evidence-needs-mtg-report.pdf (accessed July 7, 2022).

[7] World Health Organization. Declaration of the Sixth Ministerial Conference on Environment and Health: Annex 1. Compendium of Possible Actions to Advance the Implementation of the Ostrava Declaration. World Health Organization; Regional Office for Europe (2017). Available online at: https://apps.who.int/iris/handle/10665/347249 (accessed July 5, 2022).

[8] Fazzo L Minichilli F Santoro M Ceccarini A Della Seta M Bianchi F. Hazardous waste and health impact: a systematic review of the scientific literature. Environ Health. (2017) 16:107. 10.1186/s12940-017-0311-8 29020961 PMC5637250

[9] García-Pérez J Morales-Piga A Gómez-Barroso D Tamayo-Uria I Pardo Romaguera E López-Abente G. Risk of bone tumors in children and residential proximity to industrial and urban areas: new findings from a case-control study. Sci Total Environ. (2017) 579:1333–42. 10.1016/j.scitotenv.2016.11.131 27916304

[10] Kihal-Talantikite W Zmirou-Navier D Padilla C Deguen S. Systematic literature review of reproductive outcome associated with residential proximity to polluted sites. Int J Health Geogr. (2017) 16:20. 10.1186/s12942-017-0091-y 28558782 PMC5450119

[11] Santoro M Minichilli F Pierini A Astolfi G Bisceglia L Carbone P. Congenital anomalies in contaminated sites: a multisite study in Italy. Int J Environ Res Public Health. (2017) 14:292. 10.3390/ijerph14030292 28287452 PMC5369128

[12] Kowalska M Kulka E Jarosz W Kowalski M. The determinants of lead and cadmium blood levels for preschool children from industrially contaminated sites in Poland. Int J Occup Med Environ Health. (2018) 31:351–9. 10.13075/ijomeh.1896.01153 29072712

[13] Salerno C Marciani P Esposito A Palin LA. Mortality in the district of Ghemme and Cavaglio d'agogna, site of an urban waste landfill. Ig Sanita Pubbl. (2018) 74:35–48. 29734321

[14] Tlotleng N Kootbodien T Wilson K Made F Mathee A Ntlebi V. Prevalence of respiratory health symptoms among landfill waste recyclers in the city of Johannesburg, South Africa. Int J Environ Res Public Health. (2019) 16:4277. 10.3390/ijerph16214277 31689929 PMC6862197

[15] Mazzucco W Tavormina E Macaluso M Marotta C Cusimano R Alba D. Do emissions from landfill fires affect pregnancy outcomes? A retrospective study after arson at a solid waste facility in Sicily. BMJ Open. (2019) 9:e027912. 10.1136/bmjopen-2018-027912 31278100 PMC6615803

[16] Forte IM Indovina P Costa A Iannuzzi CA Costanzo L Marfella A. Blood screening for heavy metals and organic pollutants in cancer patients exposed to toxic waste in southern Italy: a pilot study. J Cell Physiol. (2020) 235:5213–22. 10.1002/jcp.29399 31838757

[17] Made F Ntlebi V Kootbodien T Wilson K Tlotleng N Mathee A. Illness, Self-rated health and access to medical care among waste pickers in landfill sites in Johannesburg, South Africa. Int J Environ Res Public Health. (2020) 17:2252. 10.3390/ijerph17072252 32230743 PMC7177792

[18] Narduzzi S Fantini F Blasetti F Rantakokko P Kiviranta H Forastiere F. Predictors of beta-hexachlorocyclohexane blood levels among people living close to a chemical plant and an illegal dumping site. Environ Health. (2020) 19:9. 10.1186/s12940-020-0562-7 31969154 PMC6977344

[19] Norsa'adah B Salinah O Naing NN Sarimah A. Community health survey of residents living near a solid waste open dumpsite in Sabak, Kelantan, Malaysia. Int J Environ Res Public Health. (2020) 17:311. 10.3390/ijerph17010311 31906421 PMC6981880

[20] Tomita A Cuadros DF Burns JK Tanser F Slotow R. Exposure to waste sites and their impact on health: a panel and geospatial analysis of nationally representative data from South Africa, 2008-2015. Lancet Planet Health. (2020) 4:e223–34. 10.1016/S2542-5196(20)30101-7 32559439 PMC7302423

[21] Abiola AO Fakolade FC Akodu BA Adejimi AA Oyeleye OA Sodamade GA. Comparison of respiratory and skin disorders between residents living close to and far from Solous landfill site in Lagos State, Nigeria. Afr J Prim Health Care Fam Med. (2021) 13:e1–7. 10.4102/phcfm.v13i1.2677 33970013 PMC8111640

[22] Vinti G Bauza V Clasen T Medlicott K Tudor T Zurbrügg C. Municipal solid waste management and adverse health outcomes: a systematic review. Int J Environ Res Public Health. (2021) 18:4331. 10.3390/ijerph18084331 33921868 PMC8072713

[23] Ibrahim MF Hod R Toha HR Mohammed Nawi A Idris IB Mohd Yusoff H. The impacts of illegal toxic waste dumping on children's health: a review and case study from Pasir Gudang, Malaysia. Int J Environ Res Public Health. (2021) 18:2221. 10.3390/ijerph18052221 33668186 PMC7956593

[24] Ngo HTT Watchalayann P Nguyen DB Doan HN Liang L. Environmental health risk assessment of heavy metal exposure among children living in an informal e-waste processing village in Viet Nam. Sci Total Environ. (2021) 763:142982. 10.1016/j.scitotenv.2020.142982 33129545

[25] Dai Q Xu X Eskenazi B Asante KA Chen A Fobil J. Severe dioxin-like compound (DLC) contamination in e-waste recycling areas: an under-recognized threat to local health. Environ Int. (2020) 139:105731. 10.1016/j.envint.2020.105731 32315892

[26] Shi J Zheng GJ Wong MH Liang H Li Y Wu Y. Health risks of polycyclic aromatic hydrocarbons via fish consumption in Haimen bay (China), downstream of an e-waste recycling site (Guiyu). Environ Res. (2016) 147:233–40. 10.1016/j.envres.2016.01.036 26897061

[27] Xue K Qian Y Wang Z Guo C Wang Z Li X. Cobalt exposure increases the risk of fibrosis of people living near E-waste recycling area. Ecotoxicol Environ Saf. (2021) 215:112145. 10.1016/j.ecoenv.2021.112145 33743401

[28] Ericson B Landrigan P Taylor MP Frostad J Caravanos J Keith J. The global burden of lead toxicity attributable to informal used lead-acid battery sites. Ann Glob Health. (2016) 82:686–99. 10.1016/j.aogh.2016.10.015 28283119

[29] Wang H Huang P Zhang R Feng X Tang Q Liu S. Effect of lead exposure from electronic waste on haemoglobin synthesis in children. Int Arch Occup Environ Health. (2021) 94:911–8. 10.1007/s00420-020-01619-1 33474627 PMC8238723

[30] Xu L Huo X Liu Y Zhang Y Qin Q Xu X. Hearing loss risk and DNA methylation signatures in preschool children following lead and cadmium exposure from an electronic waste recycling area. Chemosphere. (2020) 246:125829. 10.1016/j.chemosphere.2020.125829 31927382

[31] Cai H Xu X Zhang Y Cong X Lu X Huo X. Elevated lead levels from e-waste exposure are linked to sensory integration difficulties in preschool children. Neurotoxicology. (2019) 71:150–8. 10.1016/j.neuro.2019.01.004 30664973

[32] Zeng X Xu X Zheng X Reponen T Chen A Huo X. Heavy metals in PM2.5 and in blood, and children's respiratory symptoms and asthma from an e-waste recycling area. Environ Pollut. (2016) 210:346–53. 10.1016/j.envpol.2016.01.025 26803791

[33] Zeng X Xu X Boezen HM Vonk JM Wu W Huo X. Decreased lung function with mediation of blood parameters linked to e-waste lead and cadmium exposure in preschool children. Environ Pollut. (2017) 230:838–48. 10.1016/j.envpol.2017.07.014 28734265

[34] Lu X Xu X Zhang Y Zhang Y Wang C Huo X. Elevated inflammatory Lp-PLA2 and IL-6 link e-waste Pb toxicity to cardiovascular risk factors in preschool children. Environ Pollut. (2018) 234:601–9. 10.1016/j.envpol.2017.11.094 29223817

[35] Kim SS Xu X Zhang Y Zheng X Liu R Dietrich KN. Birth outcomes associated with maternal exposure to metals from informal electronic waste recycling in Guiyu, China. Environ Int. (2020) 137:105580. 10.1016/j.envint.2020.105580 32078870 PMC7257595

[36] Huo X Wu Y Xu L Zeng X Qin Q Xu X. Maternal urinary metabolites of PAHs and its association with adverse birth outcomes in an intensive e-waste recycling area. Environ Pollut. (2019) 245:453–61. 10.1016/j.envpol.2018.10.098 30458375

[37] Zeng X Xu X Zhang Y Li W Huo X. Chest circumference and birth weight are good predictors of lung function in preschool children from an e-waste recycling area. Environ Sci Pollut Res Int. (2017) 24:22613–21. 10.1007/s11356-017-9885-5 28808870

[38] Liu Y Huo X Xu L Wei X Wu W Wu X. Hearing loss in children with e-waste lead and cadmium exposure. Sci Total Environ. (2018) 624:621–7. 10.1016/j.scitotenv.2017.12.091 29272831

[39] Parvez SM Jahan F Brune MN Gorman JF Rahman MJ Carpenter D. Health consequences of exposure to e-waste: an updated systematic review. Lancet Planet Health. (2021) 5:e905–20. 10.1016/S2542-5196(21)00263-1 34895498 PMC8674120

[40] Fazzo L De Santis M Beccaloni E Scaini F Iavarone I Comba P. A geographic information system-based indicator of waste risk to investigate the health impact of landfills and uncontrolled dumping sites. Int J Environ Res Public Health. (2020) 17:5789. 10.3390/ijerph17165789 32785131 PMC7459911

[41] Fazzo L Belli S Minichilli F Mitis F Santoro M Martina L. Cluster analysis of mortality and malformations in the provinces of Naples and Caserta (Campania region). Ann Ist Super Sanita. (2008) 44:99–111. 18469382

[42] Martuzzi M Mitis F Bianchi F Minichilli F Comba P Fazzo L. Cancer mortality and congenital anomalies in a region of Italy with intense environmental pressure due to waste. Occup Environ Med. (2009) 66:725–32. 10.1136/oem.2008.044115 19416805

[43] Fazzo L De Santis M Mitis F Benedetti M Martuzzi M Comba P. Ecological studies of cancer incidence in an area interested by dumping waste sites in Campania (Italy). Ann Ist Super Sanita. (2011) 47:181–91. 10.4415/ANN_11_02_10 21709388

[44] Benedetti M Fazzo L Buzzoni C Comba P Magnani C Fusco M. Incidence of soft tissue sarcomas in an Italian area affected by illegal waste dumping sites. Arch Environ Occup Health. (2015) 70:154–9. 10.1080/19338244.2013.845135 24219564

[45] Pearce N. Traditional epidemiology, modern epidemiology, and public health. Am J Public Health. (1996) 86:678–83. 10.2105/AJPH.86.5.678 8629719 PMC1380476

[46] Grandjean P Budtz-Jørgensen E Keiding N Weihe P. Underestimation of risk due to exposure misclassification. Int J Occup Med Env Health. (2004) 17:131–36. 15212216

[47] Savitz DA. Interpreting Epidemiologic Evidence: Strategies for Study Design and Analysis. New York, NY: Oxford University Press (2003). 10.1093/acprof:oso/9780195108408.001.0001

[48] Checkoway H Pearce N Kriebel D. Research Methods in Occupational Epidemiology. New York, NY: Oxford University Press (2004). 10.1093/acprof:oso/9780195092424.001.0001

[49] Jurek AM Greenland S Maldonado G. How far from non-differential does exposure or disease misclassification have to be to bias measures of association away from the null? Int J Epidemiol. (2008) 37:382–85. 10.1093/ije/dym291 18184671

[50] Anselin L Xun L. Tobler's law in multivariate world. Geograp Anal. (2020) 52:494. 10.1111/gean.12237

[51] De Felip E Bianchi F Bove C Cori L D'Argenzio A D'Orsi G. Priority persistent contaminants in people dwelling in critical areas of Campania region, Italy (SEBIOREC biomonitoring study). Sci Total Environ. (2014) 487:420–35. 10.1016/j.scitotenv.2014.04.016 24797738

[52] Esposito M Cavallo S Serpe FP D'Ambrosio R Gallo P Colarusso G. Levels and cogener profiles of polychlorinated dibenzo-p-dioxins, polychlorinated dibenzofurans and dioxin-like polychlorinated biphenyls in cow's milk collected in Campania, Italy. Chemosphere. (2009) 77:1212–6. 10.1016/j.chemosphere.2009.09.011 19836049

[53] Giovannini A Ribezzi G Carideo P Ceci R Diletti G Ippoliti C. Dixons levels in breast milk of women living in Caserta and Naples: assessment of environmental risk factors. Chemosphere. (2014) 94:76–84. 10.1016/j.chemosphere.2013.09.017 24120012

[54] Rivezzi G Piscitelli P Scortichini G Giovannini A Diletti G Migliorati G. A general model of dioxin contamination in breast milk: results from a study on 94 women from the Caserta and Naples areas in Italy. Int J Res Public Health. (2013) 10:5953–70. 10.3390/ijerph10115953 24217180 PMC3863880

[55] Stura A Gangemi M Mirabelli D. Uso delle schede di dimissione ospedaliera per la stima dell'incidenza dei mesoteliomi maligni. Epidemiol Prev. (2007) 31:127–31. 18677861

[56] International Agency for Research on Cancer. IARC List of Classification by Cancer Sites With Sufficient or Limited Evidence in Humans. Vol. 1–113. Lyon: International Agency for Research on Cancer (2014).

[57] World Health Organization/United Nations Environment Programme (WHO/UNEP). State of the Science of Endocrine Disrupting Chemicals 2012 an Assessment of the State of the Science of Endocrine Disruptors Prepared by a Group of Experts for the United Nations Environment Programme and World Health Organization. Bergman A, Jerrold J, Heindel JJ, Jobling S, Karen A, Kidd KA, Zoeller RT, editors (2013). Available online at: https://apps.who.int/iris/bitstream/handle/10665/78102/WHO_HSE_PHE_IHE_2013.1_eng.pdf (acceesed Febraury 10, 2023).

[58] Benedetti M Zona A Beccaloni E Carere M Comba P. Incidence of breast, prostate, testicular, and thyroid cancer in italian contaminated sites with presence of substances with endocrine disrupting properties. Int J Environ Res Public Health. (2017) 14:355. 10.3390/ijerph14040355 28353667 PMC5409556

[59] Boberg E Lessner L Carpenter DO. The role of residence near hazardous waste sites containing benzene in the development of hematologic cancers in upstate New York. Int J Occup Med Environ Health. (2011) 24:327–38. 10.2478/s13382-011-0037-8 22002323

[60] Carpenter DO Ma J Lessner L. Asthma and infectious respiratory disease in relation to residence near hazardous waste sites. Ann N Y Acad Sci. (2008) 1140:201–8. 10.1196/annals.1454.000 18991918

[61] World Health Organization. Effects of Air Pollution on Children's Health and Development. A Review of the Evidence. Copenhagen: World Health Organization, Regional Office for Europe (2005). Available online at: http://www.euro.who.int/__data/assets/pdf_file/0010/74728/E86575.pdf (accessed July 15, 2022).

[62] World Health Organization. Inheriting a Sustainable World? Atlas on Children's Health the Environment. Geneva: World Health Organization (2017). Available online at: https://www.who.int/publications/i/item/9789241511773 (accessed February 10, 2023).

[63] García-Pérez J López-Abente G Gómez-Barroso D Morales-Piga A Romaguera EP Tamayo I. Childhood leukemia and residential proximity to industrial and urban sites. Environ Res. (2015) 140:542–53. 10.1016/j.envres.2015.05.014 26025512

[64] Torchin H Ancel PH. Epidémiologie et facteurs de risque de la prématurité. J Gynecol Obstet Biol Reprod. (2016) 45:1213–30. 10.1016/j.jgyn.2016.09.013 27789055

[65] March March of Dimes PMNCH Save the Children WHO. Born Too Soon: The Global Action Report on Preterm Birth. Howson CP, Kinney MV, Lawn JE, editors. Geneva: World Health Organization (2012).

[66] Chehade H Simeoni U Guignard JP Boubred F. Preterm birth: long term cardiovascular and renal consequences. Curr Pediatr Rev. (2018) 14:219–26. 10.2174/1573396314666180813121652 30101715 PMC6416185

[67] Parkinson JR Hyde MJ Gale C Santhakumaran S Modi N. Preterm birth and the metabolic syndrome in adult life: a systematic review and meta-analysis. Pediatrics. (2013) 131:e1240–63. 10.1542/peds.2012-2177 23509172

[68] de Jong F Monuteaux MC van Elburg RM Gillman MW Belfort MB. Systematic review and meta-analysis of preterm birth and later systolic blood pressure. Hypertension. (2012) 59:226–34. 10.1161/HYPERTENSIONAHA.111.181784 22158643 PMC3266458

[69] Markopoulou P Papanikolaou E Analytis A Zoumakis E Siahanidou T. Preterm birth as a risk factor for metabolic syndrome and cardiovascular disease in adult life: a systematic review and meta-analysis. J Pediatr. (2019) 210:69–80.e5. 10.1016/j.jpeds.2019.02.041 30992219

[70] Hill B. The environment and disease: association or causation? Proc R Soc Med. (1965) 58:295–300. 10.1177/003591576505800503 14283879 PMC1898525

[71] Merrill RM. Environmental Epidemiology: Principles Methods. Sudbury, MA: Jones & Bartlett Publishers (2008).

[72] Morgenstern H. Ecologic studies. In:Rothman KJ Greenland S Lash TL, editors. Modern Epidemiology, 3rd edition. Philadelphia, PA: Lippincott Williams & Wilkins (2008). p. 511–31.

[73] International Agency for Research on Cancer,. IARC Monographs on the Identification of Carcinogenic Hazards to Humans. Preamble (2019). Available online at: https://monographs.iarc.who.int/wp-content/uploads/2019/07/Preamble-2019.pdf (accessed July 15, 2022).

[74] Hoffman RE. The use of epidemiologic data in the courts. Am J Epidemiol. (1984) 120:190–202. 10.1093/oxfordjournals.aje.a113881 6465117

[75] Ginzburg HM. Use and misuse of epidemiologic data in the courtroom: defining the limits of inferential and particularistic evidence in mass tort litigation. Am J Law Med. (1986) 12:423–39 10.1017/S0098858800009758 3451678

[76] Kune R Kune G. Proof of cancer causation and expert evidence: bringing science to the law and the law to science. J Law Med. (2003) 11:112–21 14526731

[77] Lagiou P Adami HO Trichopoulos D. Causality in cancer epidemiology. Eur J Epidemiol. (2005) 20:565–74. 10.1007/s10654-005-7968-y 16119428

[78] Douglas CE Davis RM Beasley JK. Epidemiology of the third wave of tobacco litigation in the United States, 1994-2005. Tob Control. (2006) 15 (Suppl. 4):iv9–16. 10.1136/tc.2006.016725 17130629 PMC2563581

[79] Barone-Adesi F Bruno C Calisti R ChelliniE Comba P Consonni D. Effects of asbestos on human health. Document of the Italian epidemiological association (AIE). Epidemiol Prev. (2020) 44:327–38. 10.19191/EP20.5-6.A001.064 33506658

[80] Fazzo L Bianchi F Carpenter D Martuzzi M Comba P. Hazardous waste: a challenge for public health. Public Health Panorama. (2017) 3:247–52.

[81] Marsili D Fazzo L Iavarone I Comba P. Communications plans in contaminated area sas prevention tools for informed policy. Public Health Panorama. (2017) 3:261–7.

[82] Sarigiannis D. Assessing the impact of hazardous waste on children's health: the exposome paradigm. Environ Res. (2017) 158:531–41. 10.1016/j.envres.2017.06.031 28711809

[83] Sarigiannis DA Karakitsios SP. Addressing complexity of health impact assessment in industrially contaminated sites via exposome paradigm. Epidemiol Prev. (2018) 42:37–48. 10.19191/EP18.5-6.S1.P037.086 30322234

[84] Hoek G Ranzi A Alimehmeti I Ardeleanu ER Arrebola JP Avila P et al. A review of exposure assessment methods for epidemiological studies of health effects related to industrially contaminated sites. Epidemiol Prev. (2018) 42:21–36. 10.19191/EP18.5-6.S1.P021.085 30322233

