# Supplementary material for: The health impact of hazardous waste landfills and illegal dumps contaminated sites: An epidemiological study at ecological level in Italian Region
Source: Front Public Health. 2023 Feb 27;11:996960. doi: 10.3389/fpubh.2023.996960 (PMC10010672; doi:10.3389/fpubh.2023.996960)
Supplement: Supplementary file 1 [file Table_1.docx]

Table S1: Population at study (2019 Census), by municipality (a) and Municipal waste Risk Indicator (MRI) class (b).

| **Table S1.a** |  |  |  |  |
| --- | --- | --- | --- | --- |
| **Municipality** | **Males** | **Females** | **Total** | **MRI Class** |
| Arzano | 16547 | 17209 | 33756 | 1 |
| Calvizzano | 5811 | 6261 | 12072 | 1 |
| Carinaro | 3441 | 3602 | 7043 | 1 |
| Casapesenna | 3402 | 3495 | 6897 | 1 |
| Casavatore | 9035 | 9511 | 18546 | 1 |
| Cesa | 4636 | 4627 | 9263 | 1 |
| Frattamaggiore | 13858 | 14877 | 28735 | 1 |
| Frattaminore | 7816 | 8035 | 15851 | 1 |
| Frignano | 4507 | 4644 | 9151 | 1 |
| Grumo Nevano | 8812 | 8848 | 17660 | 1 |
| Marano di Napoli | 28219 | 29913 | 58132 | 1 |
| Parete | 5896 | 6012 | 11908 | 1 |
| San Cipriano d'Aversa | 6789 | 6801 | 13590 | 1 |
| San Marcellino | 7189 | 7141 | 14330 | 1 |
| Sant'Antimo | 16337 | 17003 | 33340 | 1 |
| Succivo | 4323 | 4350 | 8673 | 1 |
| Teverola | 7161 | 7385 | 14546 | 1 |
| Trentola Ducenta | 9724 | 10046 | 19770 | 1 |
| Villa di Briano | 3607 | 3573 | 7180 | 1 |
| Villa Literno | 6290 | 5928 | 12218 | 1 |
| Afragola | 30886 | 31922 | 62808 | 2 |
| Aversa | 24703 | 26641 | 51344 | 2 |
| Casal di Principe | 10684 | 10822 | 21506 | 2 |
| Casaluce | 4703 | 4955 | 9658 | 2 |
| Casandrino | 7084 | 6477 | 13561 | 2 |
| Crispano | 5931 | 6063 | 11994 | 2 |
| Gricignano di Aversa | 6275 | 6253 | 12528 | 2 |
| Lusciano | 7700 | 8081 | 15781 | 2 |
| Orta di Atella | 13748 | 13807 | 27555 | 2 |
| Qualiano | 12512 | 12706 | 25218 | 2 |
| Sant'Arpino | 7389 | 7442 | 14831 | 2 |
| Cardito | 10786 | 11082 | 21868 | 3 |
| Casoria | 36284 | 38665 | 74949 | 3 |
| Melito di Napoli | 17656 | 18192 | 35848 | 3 |
| Mugnano di Napoli | 16883 | 17922 | 34805 | 3 |
| Villaricca | 14932 | 15860 | 30792 | 3 |
| Caivano | 18316 | 18910 | 37226 | 4 |
| Giugliano in Campania | 58703 | 59873 | 118576 | 4 |

| **Table S1.b** |  |  |  |
| --- | --- | --- | --- |
| **Totals by MRI Class** | **Males** | **Females** | **Total** |
| 1 | 173400 | 179261 | 352661 |
| 2 | 131615 | 135169 | 266784 |
| 3 | 96541 | 101721 | 198262 |
| 4 | 77019 | 78783 | 155802 |
| **Total** | **478575** | **494934** | **973509** |
