# Supplementary material for: The health impact of hazardous waste landfills and illegal dumps contaminated sites: An epidemiological study at ecological level in Italian Region
Source: Front Public Health. 2023 Feb 27;11:996960. doi: 10.3389/fpubh.2023.996960 (PMC10010672; doi:10.3389/fpubh.2023.996960)
Supplement: Supplementary file 2 [file Table_2.docx]

Table S2. Class 1 of MRI: Mortality and hospitalization (2008-2019), with respect to the regional population, by gender. General population (all ages).

| **DISEASES** | **HOSPITALIZATION** | | **MORTALITY** | |
| --- | --- | --- | --- | --- |
|  | **MEN** | **WOMEN** | **MEN** | **WOMEN** |
|  | **SHR (90%CI)** | **SHR (90%CI)** | **SMR (90%CI)** | **SMR (90%CI)** |
| Malignant neoplasm (MN) of liver | 128 (120-136) | 131 (119-144) | 138 (128-148) | 144 (131-160) |
| MN of testis | 85 (75-98) |  | 71 (35-147) |  |
| MN of breast | 81 (56-117) | 97 (94-100) | 112 (61-207) | 104 (97-111) |
| MN of bladder | 117 (112-122) | 108 (99-118) | 131 (120-143) | 109 (90-134) |
| Non Hodgkin Lymphoma | 100 (92-108) | 105 (95-116) | 90 (76-105) | 109 (92-129) |

Legend: SHR: Standardized Hospitalization Ratio; SMR: Standardized Hospitalization Ratio; CI: Confidence Interval
