# Supplementary material for: The health impact of hazardous waste landfills and illegal dumps contaminated sites: An epidemiological study at ecological level in Italian Region
Source: Front Public Health. 2023 Feb 27;11:996960. doi: 10.3389/fpubh.2023.996960 (PMC10010672; doi:10.3389/fpubh.2023.996960)
Supplement: Supplementary file 3 [file Table_3.docx]

Table S3: Class 1 of MRI: hospitalization (2008-2019), with respect to regional population, 0-19 years class. Males and females combined.

| **DISEASES** | **SHR (90%CI)** |
| --- | --- |
| All malignant tumours | 90 (82-100) |
| Leukemias | 80 (63-100) |

Legend: SHR: Standardized Hospitalization Ratio; SMR: Standardized Hospitalization Ratio; CI: Confidence Interval
