# Supplementary material for: The health impact of hazardous waste landfills and illegal dumps contaminated sites: An epidemiological study at ecological level in Italian Region
Source: Front Public Health. 2023 Feb 27;11:996960. doi: 10.3389/fpubh.2023.996960 (PMC10010672; doi:10.3389/fpubh.2023.996960)
Supplement: Supplementary file 4 [file Table_4.docx]

Table S4. Class 1 of MRI, with respect to the regional population. Prevalence at birth (2003-2017). Males and femaled combined.

| **LOW BIRTH WEIGHT** | | | **PRETERM BIRTH** | | |
| --- | --- | --- | --- | --- | --- |
| **Obs** | **% on born alives** | **RP (90%CI)** | **Obs** | **% on born alives** | **RP (90%CI)** |
| 538 | 3.76 | 1.10 (1.2-1.18) | 885 | 5.82 | 0.96 (0.91-1.02) |

Legend: RP: Ratio of Prevalence; CI: Confidence Interval
