# Supplementary figures and images for: The health impact of hazardous waste landfills and illegal dumps contaminated sites: An epidemiological study at ecological level in Italian Region
Source: Front Public Health. 2023 Feb 27;11:996960. doi: 10.3389/fpubh.2023.996960 (PMC10010672; doi:10.3389/fpubh.2023.996960)

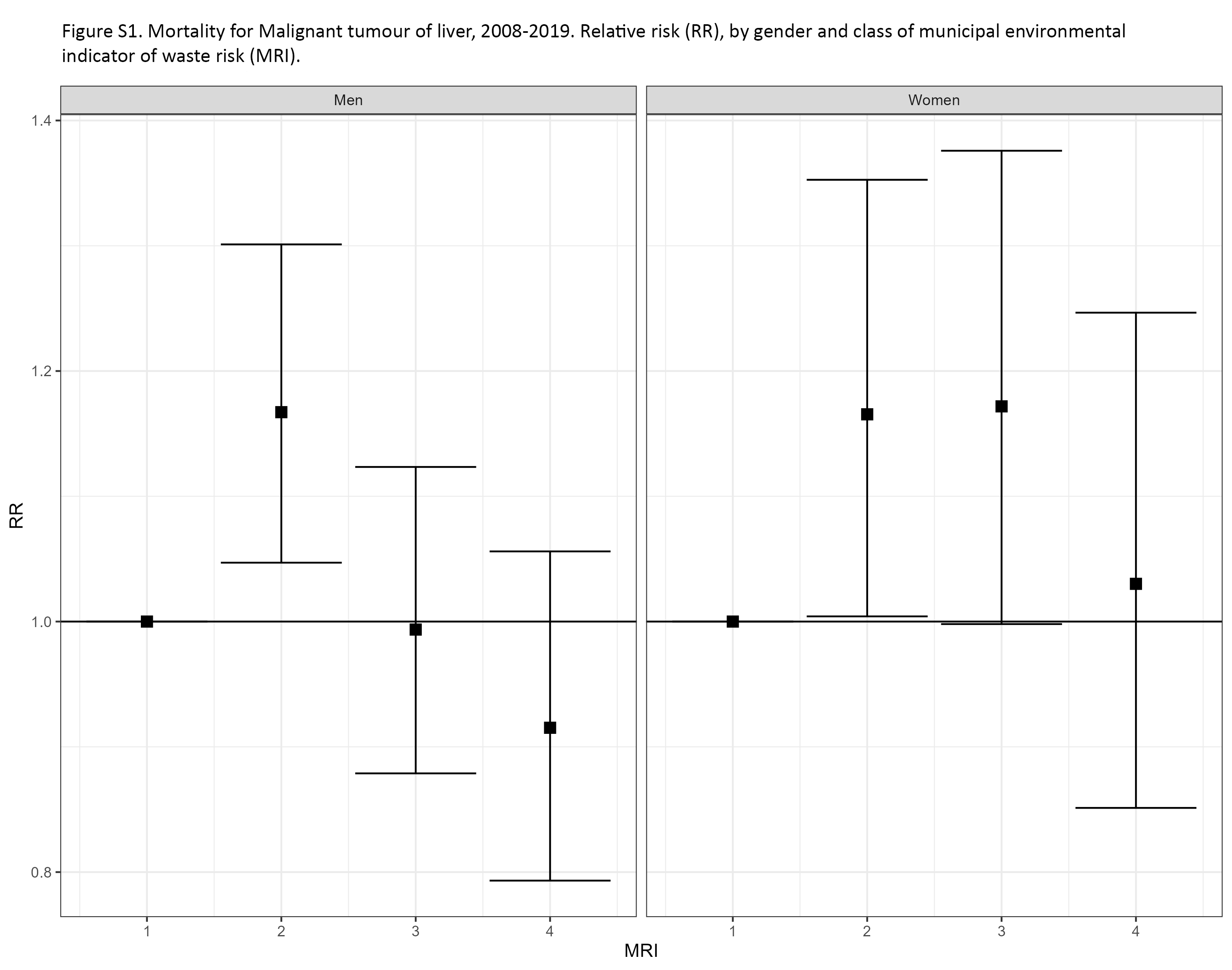

Supplement: Supplementary file 5 [file Data_Sheet_1.zip › pngNC/FigS1.png]

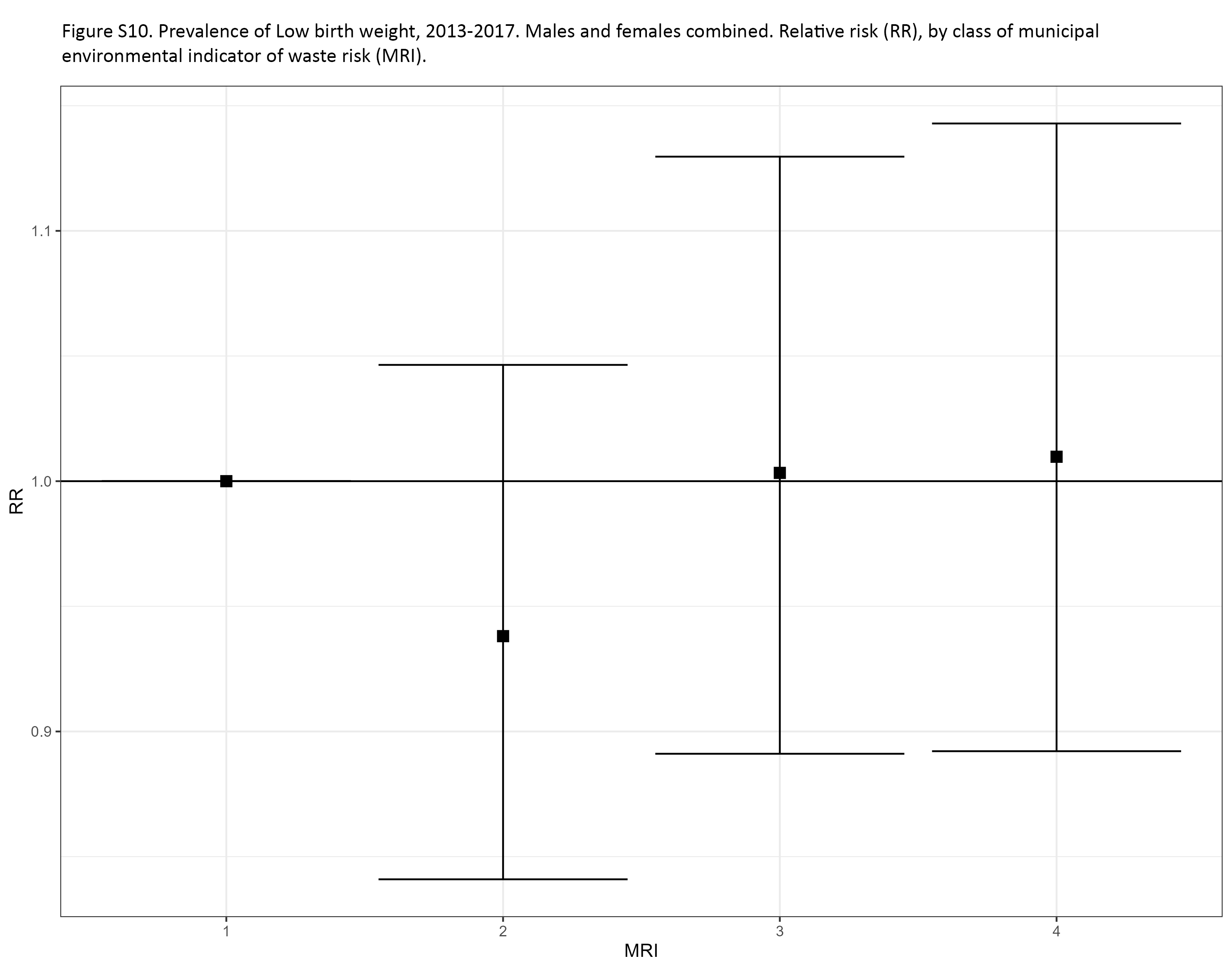

Supplement: Supplementary file 5 [file Data_Sheet_1.zip › pngNC/FigS10.png]

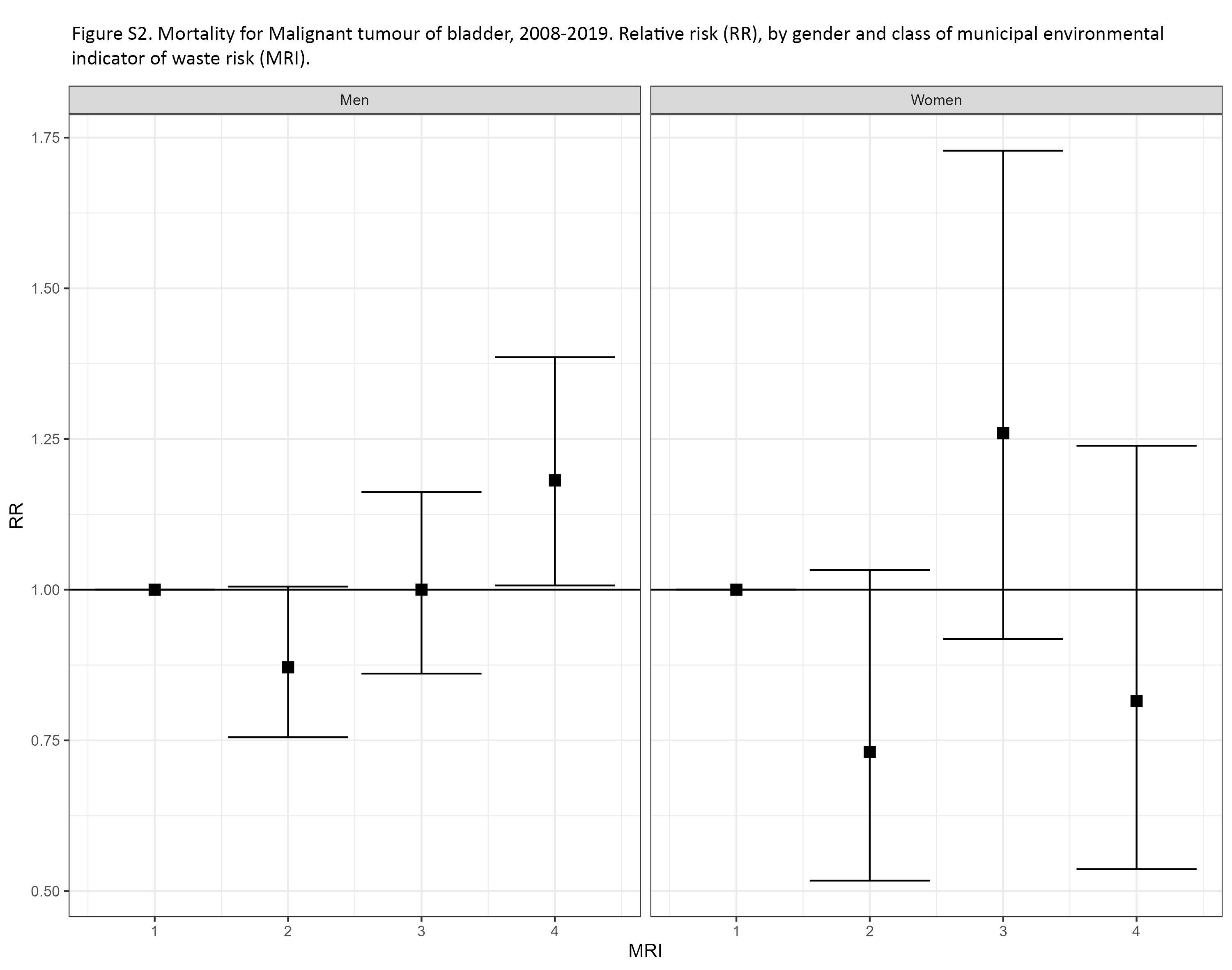

Supplement: Supplementary file 5 [file Data_Sheet_1.zip › pngNC/FigS2.png]

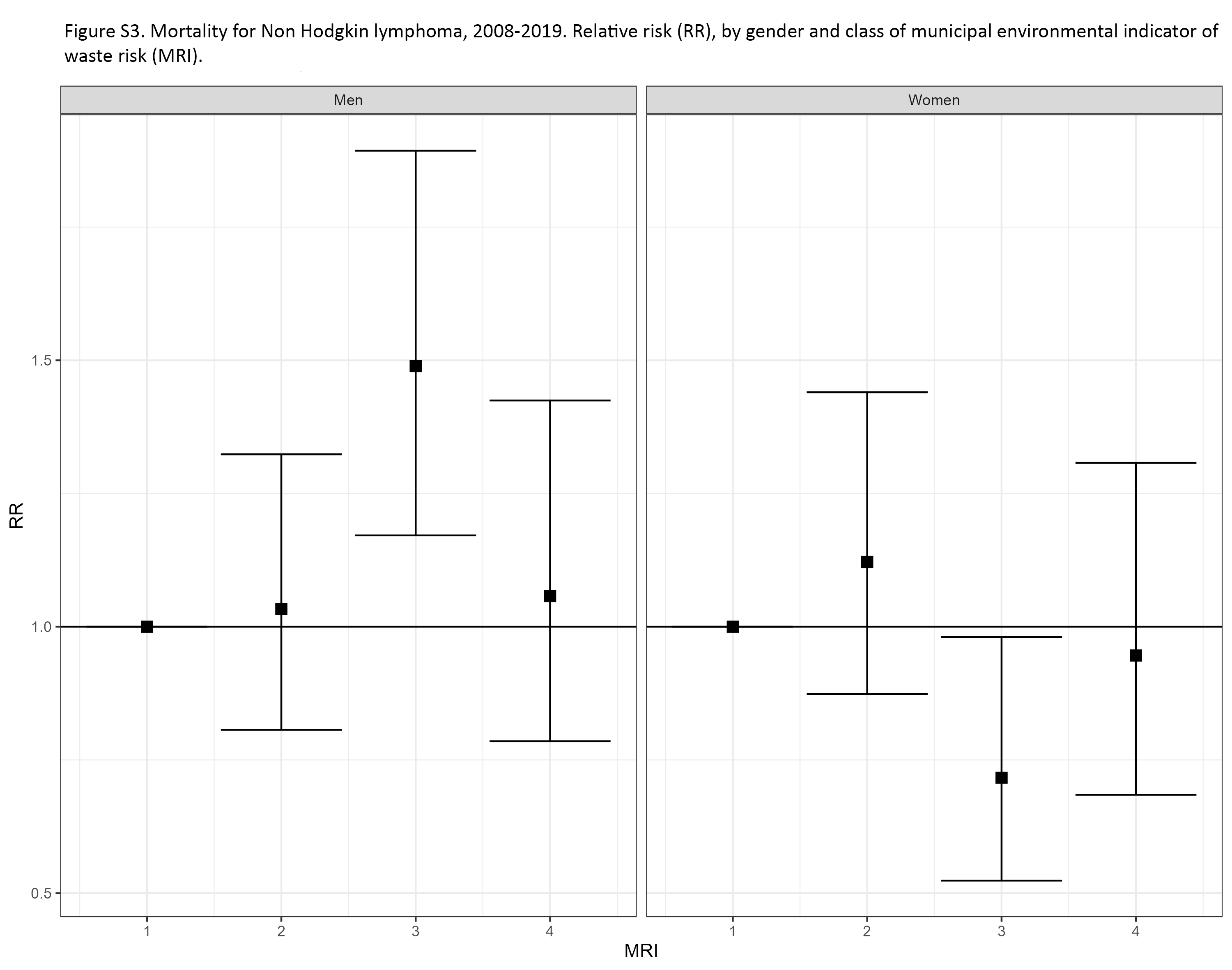

Supplement: Supplementary file 5 [file Data_Sheet_1.zip › pngNC/FigS3.png]

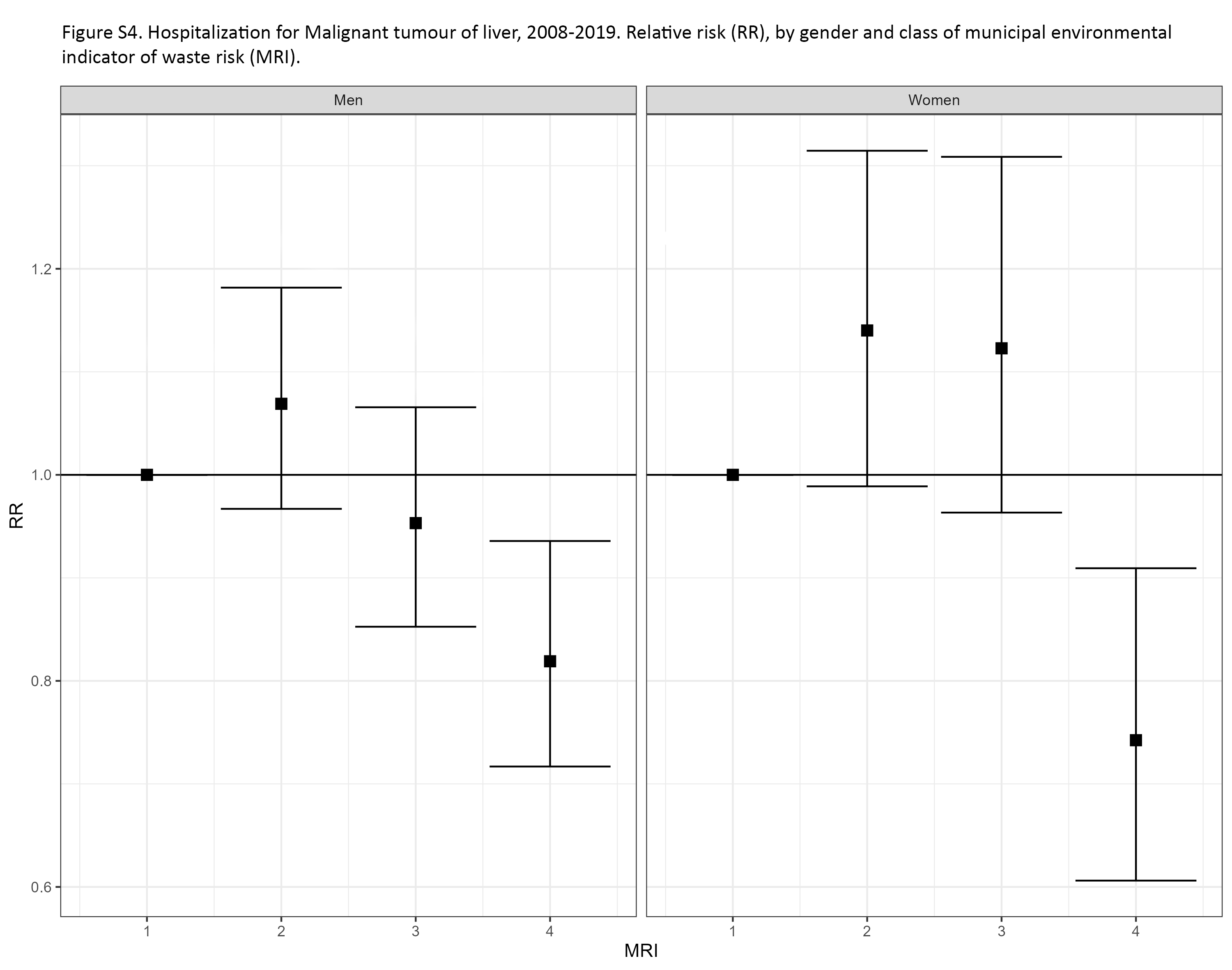

Supplement: Supplementary file 5 [file Data_Sheet_1.zip › pngNC/FigS4.png]

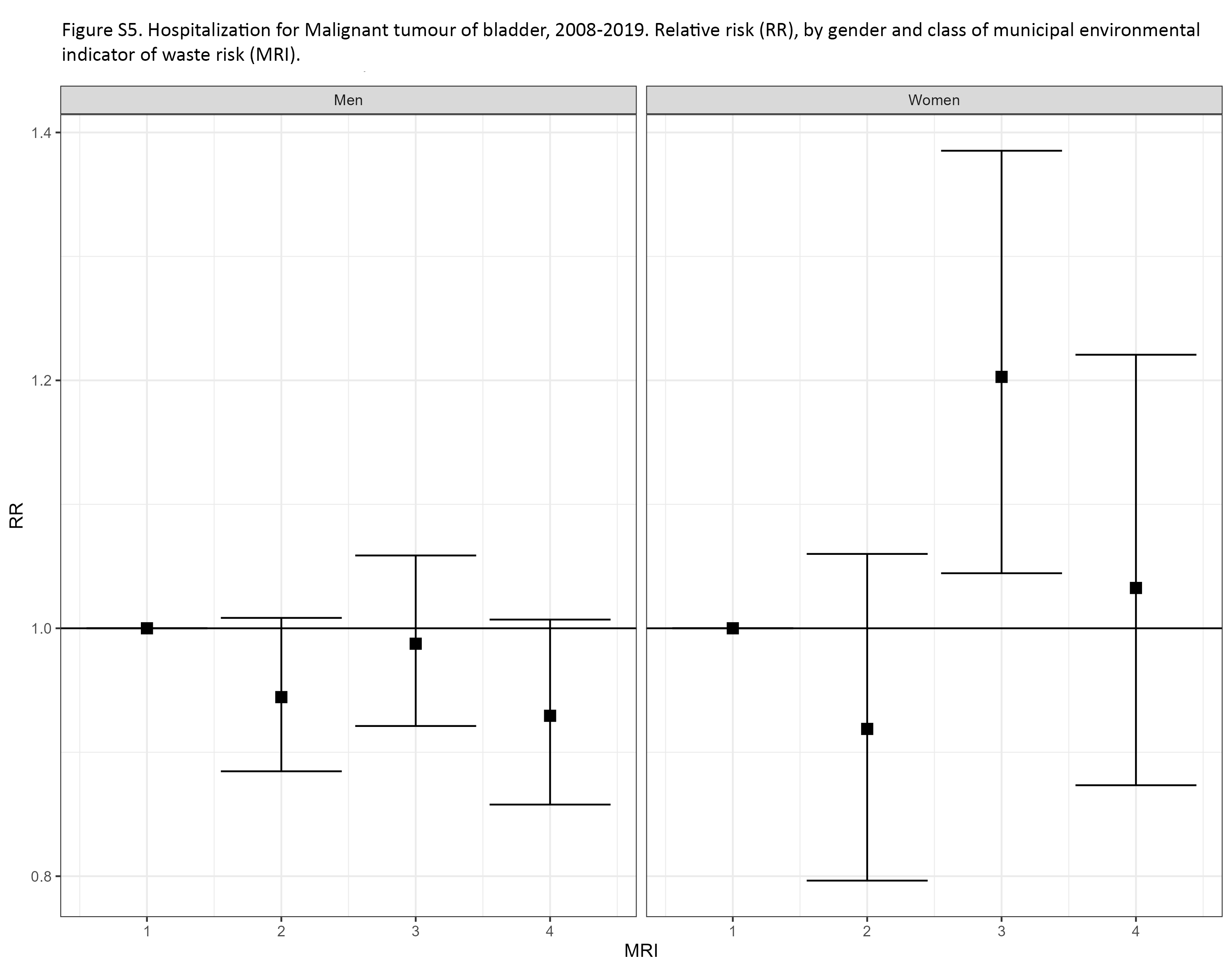

Supplement: Supplementary file 5 [file Data_Sheet_1.zip › pngNC/FigS5.png]

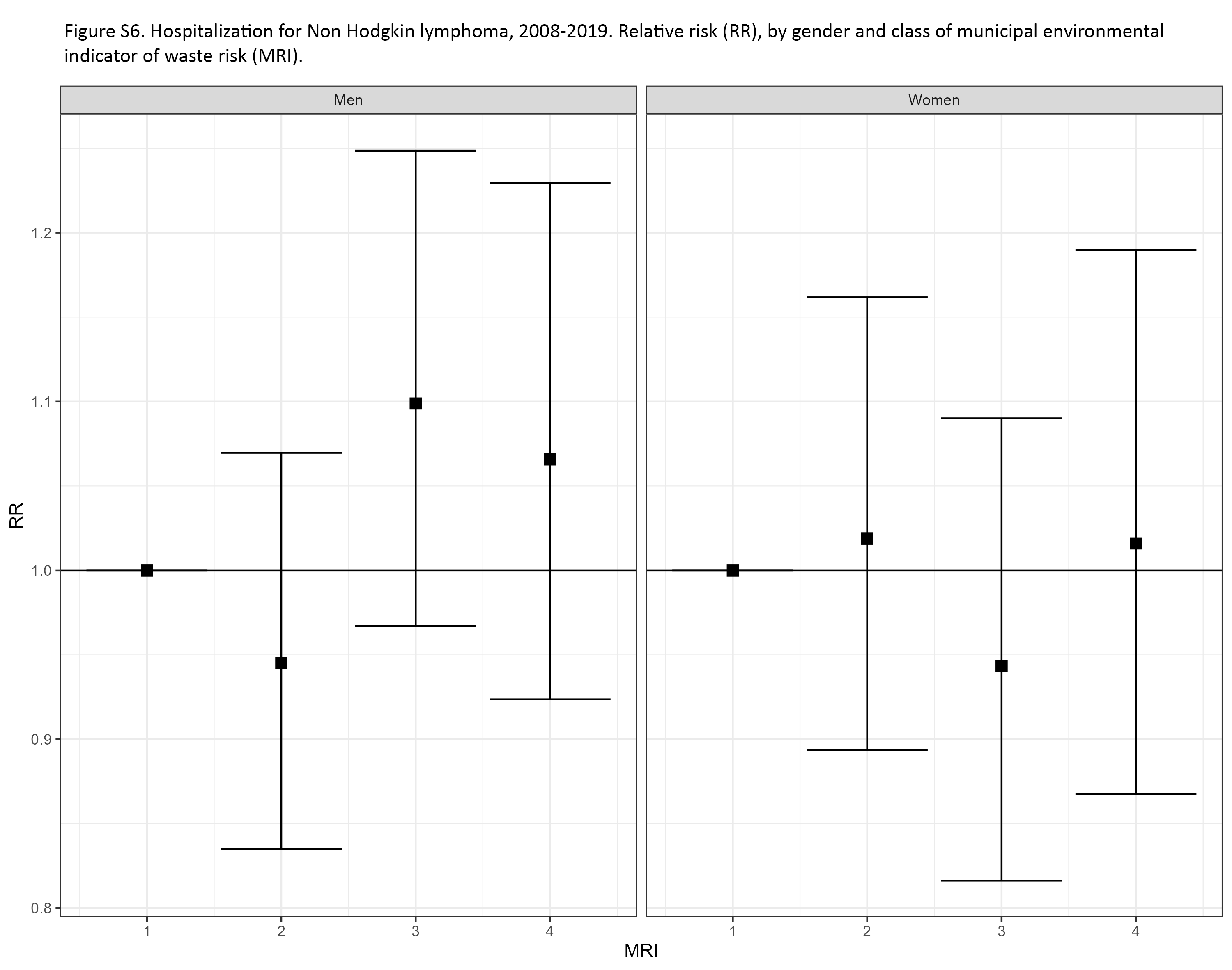

Supplement: Supplementary file 5 [file Data_Sheet_1.zip › pngNC/FigS6.png]

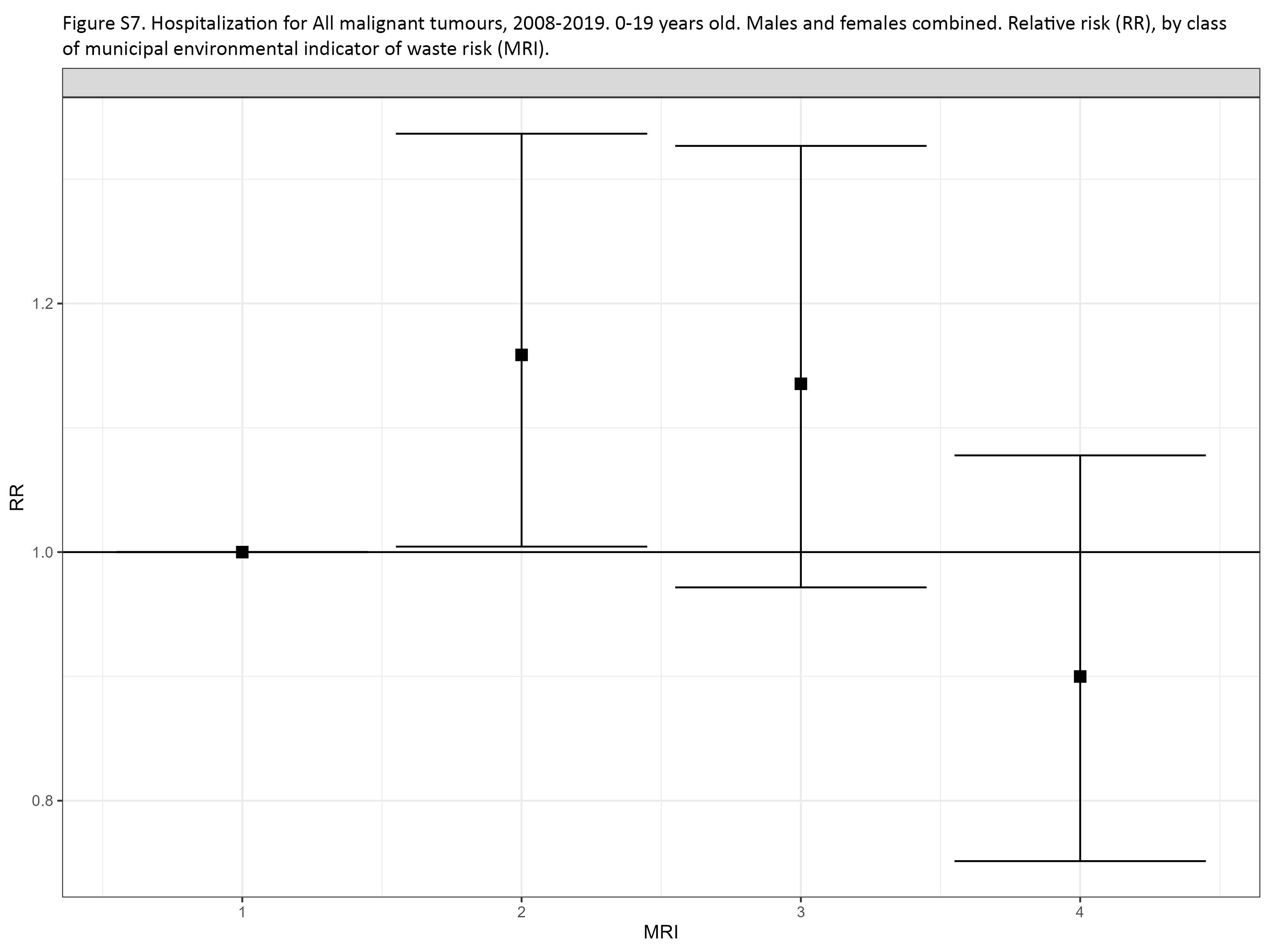

Supplement: Supplementary file 5 [file Data_Sheet_1.zip › pngNC/figS7.png]

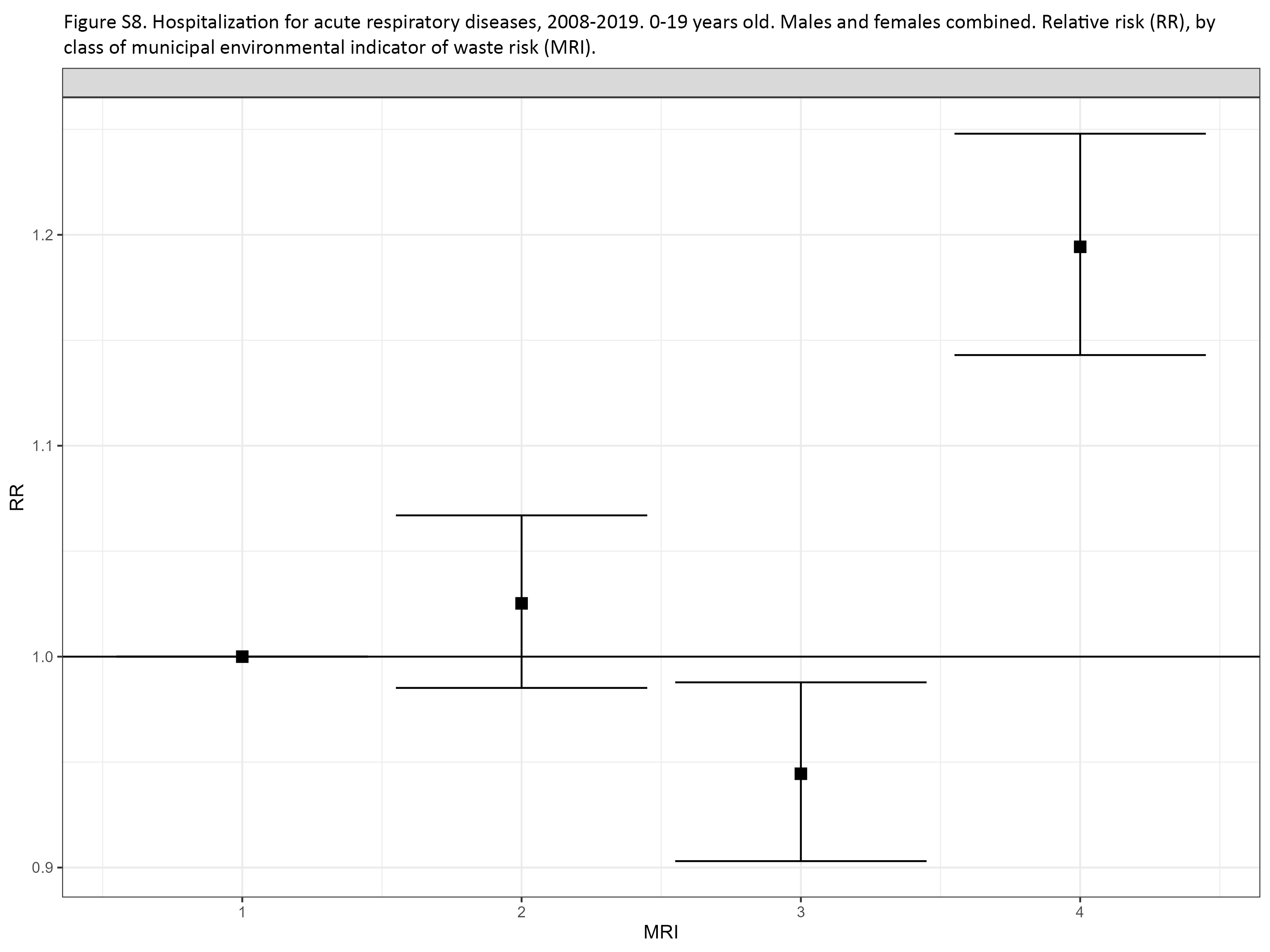

Supplement: Supplementary file 5 [file Data_Sheet_1.zip › pngNC/FigS8.png]

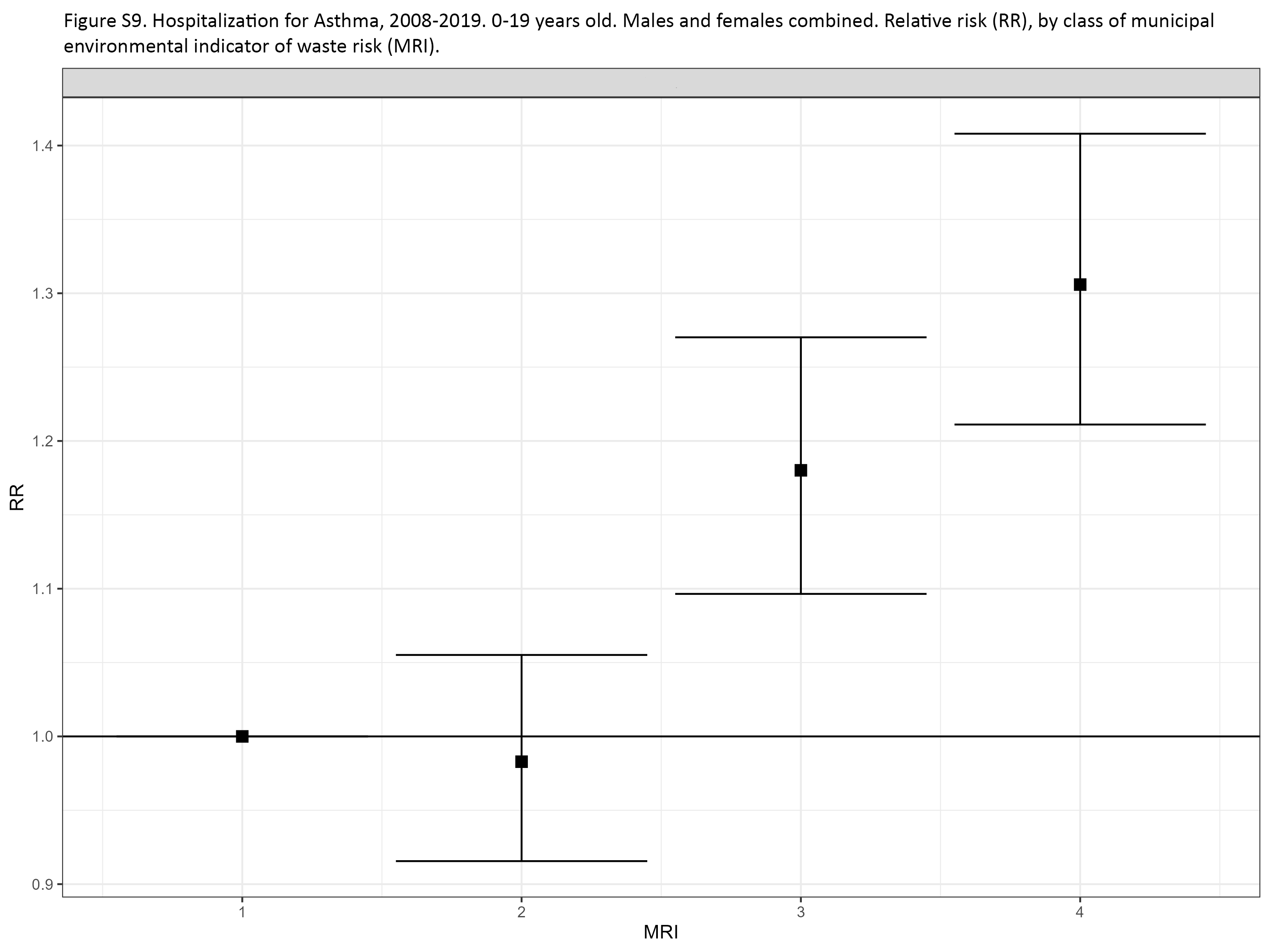

Supplement: Supplementary file 5 [file Data_Sheet_1.zip › pngNC/FigS9.png]
